# Monocyte adaptations in patients with obesity during a 1.5 year lifestyle intervention

**DOI:** 10.3389/fimmu.2022.1022361

**Published:** 2022-11-17

**Authors:** Eline S. van der Valk, Daniël S. Mulder, Tessa Kouwenhoven, Nicole M. A. Nagtzaam, Elisabeth F. C. van Rossum, Willem A. Dik, Pieter J. M. Leenen

**Affiliations:** ^1^ Obesity Center Centrum Gezond Gewicht (CGG), Erasmus Medical Center (MC), University Medical Center Rotterdam, Rotterdam, Netherlands; ^2^ Division of Endocrinology, Department of Internal Medicine, Erasmus Medical Center (MC), Rotterdam, Netherlands; ^3^ Department of Immunology, Erasmus Medical Center (MC), University Medical Center Rotterdam, Rotterdam, Netherlands; ^4^ Occupational Health and Lifestyle, VitAll, Rotterdam, Netherlands; ^5^ Laboratory Medical Immunology, Department of Immunology, Erasmus Medical Center (MC), University Medical Center Rotterdam, Rotterdam, Netherlands

**Keywords:** monocytes, classical monocytes, intermediate monocytes, non-classical monocytes, obesity, monocyte activation phenotype, combined lifestyle intervention

## Abstract

**Background:**

Obesity is associated with chronic, low-grade inflammation, which is reflected in altered peripheral blood monocyte characteristics. The aim of this study was to analyze the monocyte subset composition (classical (CM), intermediate (IM) and non-classical monocytes (NCM)), and their inflammatory marker profile (CD14, CD16, CD36, CD45, CD64, CD300e, HLA-DR) in individuals with obesity during a 1.5 year combined lifestyle intervention (CLI), comprising healthy nutrition, increased exercise and behavioral changes.

**Methods:**

We analyzed monocyte subset counts and immunophenotypes in 73 individuals with obesity, and associated these to baseline body mass index (BMI) and waist circumference (WC). The measurements were repeated after 10 weeks and at the end of the intervention (1.5 years).

**Results:**

Generally, monocyte subset counts were not associated to BMI or WC at baseline, neither did monocyte counts change during the 1.5 year CLI. Immunophenotypically, higher baseline BMI and WC were associated to lower CD14 and higher CD300e expression by all subsets. During CLI there were remarkable changes in marker profiles: expression of CD14, CD36, CD45 and CD64 significantly decreased in CM and IM, as did CD16 (IM and NCM) (p<0.05). CD300e initially decreased after 10 weeks, but increased sharply at 1.5 years (all subsets). We observed no consistent associations between changes in monocyte characteristics and anthropometric changes.

**Conclusion:**

A 1.5 year CLI in individuals with obesity mediates persistent immunophenotypic adaptations related to cellular activation in blood monocytes, whereas changes in subset distribution are limited. Lifestyle-induced changes in the inflammatory profile of monocytes differ from the ‘less-severe-obesity’-phenotype, suggesting a novel, ‘post-weight-loss’ monocyte setpoint.

## Introduction

The prevalence of obesity, defined as a body mass index (BMI) higher than 30 kg/m^2^, has been growing consistently, and worldwide there are currently more deaths linked to overweight and obesity than to underweight (1). Obesity represents an important risk factor for various types of non-communicable diseases such as cancer, cardiovascular diseases and depression. This may be partly attributed to the chronic, low-grade, inflammatory nature of obesity (2).

Along with the increase in adipocyte size and numbers, metabolic stress within the adipose tissue triggers production of inflammatory molecules that facilitate recruitment of monocytes and activation of adipose tissue macrophages (2–4). The activated adipose tissue macrophages subsequently produce additional inflammatory mediators that further enhance tissue inflammation (5, 6). Meanwhile, the activation of macrophages in patients with obesity is reflected in raised circulating levels of inflammatory mediators, such as cytokines and soluble CD163 (sCD163) (7–9).

Monocytes are central cellular elements in innate immune responses and as such involved in homeostasis, immune defense, and tissue repair (10, 11). The circulating monocyte population consists of three main subsets that differ in phenotype and function, and that are referred to as ‘classical monocytes’ (CM, CD14++CD16-), ‘intermediate monocytes’ (IM, CD14++CD16+) and ‘non-classical monocytes’ (NCM, CD14+CD16++) (10). Monocytes develop in bone marrow and are released in blood as CM, which subsequently mature into IM and NCM (12). In healthy humans in steady state, around 85% of all circulating monocytes are CM, 5% are IM and 10% are NCM (10, 11). Acute or chronic inflammatory conditions mediate a change in monocyte subset distribution, in general causing an increase in CD16-expressing subsets (10, 11). In addition, the expression of multiple immunophenotypic markers alters, indicative of monocyte activation (13).

Current literature shows that severe obesity, as compared to the lean state, is characterized by a higher number of peripheral blood monocytes, a relative increase in CD16+ monocyte subsets, and increased levels of inflammatory surface markers by all subsets (14–16). Also, monocytes are chronically activated as indicated by NF-κB nuclear translocation and increased expression of pro-inflammatory cytokines (17–19). Various previous studies have investigated whether short-term lifestyle interventions, such as energy-restricted diets with or without high protein intake (14, 20, 21) or exercise (22) can lead to changes in monocyte subset distribution. Small and inconsistent changes were found, which may depend on the diverse nature of the interventions or their duration, which varied from 6-16 weeks. As obesity is a chronic, progressive disease that is on the long term often resilient to short term weight loss interventions, it is highly relevant to obtain data of the longer term, which are currently lacking. Also, it becomes increasingly clear that a lifestyle intervention for obesity should include multiple aspects, consisting of nutritional advice, physical activity as well as structural behavioral changes, instead of any of these components alone (23). In such a combined lifestyle intervention (CLI) the beneficial effects extend beyond weight loss alone and lead to a multitude of beneficial effects (24)including relevant effects on systemic T-cell activation and an increase in regulatory T-cells, which occur independently of weight loss (7). However, how CLI affects the monocyte compartment in obesity over the longer term is unclear so far.

Therefore, in the current study we examine individuals with obesity for peripheral blood monocyte counts, subset distribution and activation profiles as indicated by immunophenotypic marker expression. First, we assess whether these monocyte characteristics are associated with clinical, obesity-related parameters at baseline. Subsequently, we follow a group of patients with obesity undergoing a CLI, to observe which of the aforementioned monocyte parameters change during the intervention and whether these changes are related to changes in body composition.

## Methods

### Subjects

Patients with obesity were enrolled in the lifestyle intervention program at Erasmus MC, University Medical Center Rotterdam after referral by their primary care physician for obesity. Inclusion criteria were: BMI ≥30 kg/m^2^ in combination with at least one of the following co-morbidities: diabetes mellitus type 2, insulin resistance (based on HOMA-IR, computed as glucose (mmol/L) x insulin (µIU/mL)/22.5), dyslipidemia, hypertension, arthrosis, sleep apnea syndrome, liver steatosis or polycystic ovarian syndrome. For diagnosis of the metabolic syndrome the joint interim statement criteria were used (25). Exclusion criteria: not proficient in the Dutch language, intellectual disability, inability to participate in physical exercise, underlying causes of obesity that require different treatment (such as genetic or endocrine disorders), insufficient motivation, severe psychopathology that requires different therapy and a current pregnancy wish. Before the start of the program all medication with potential weight-gaining side effects was optimized.

The study was approved by the local medical ethical committee (MEC2012–257).

### Intervention

The aim of the intervention was to adapt to a lifestyle that participants could maintain on the long term, and comprised, in brief (1): a healthy diet according to the Dutch guidelines for healthy nutrition (26) (without a preceding hypocaloric phase), (2) increasing exercise by increasing both aerobic and anaerobic activities, and (3) improvement in mental status with a cognitive behavioral therapy-based approach.

This lifestyle was taught either *via* small groups (Combined Lifestyle Intervention+, CLI+) or a blended care program with a partly online intervention (E-Health, EHE), according to the participants’ preferences. In the first group patients received group therapy that was led by a psychologist, a dietitian, and a physiotherapist. In the first 10 weeks, group meetings were weekly and after 10 weeks the meetings gradually tapered, until 1.5 years, as described elsewhere (24).

In the second (EHE) group the advices were given in a blended care program that included 5 multidisciplinary group sessions and an online, web-based intervention, given over the time span of 26 weeks, followed by 3 monthly follow-up meetings, as previously described (27). As the content and evaluation moments of the lifestyle intervention did not differ and numbers were small, data from the two groups were combined in all analyses.

### Evaluation moments

Patients had evaluation moments before the start of the program, after 10 weeks (T1) and after 1.5 years (T2). Then, patients were seen by a physician and routine clinical assessments were performed, including anthropometric measurements, and fasting blood samples (including glucose levels, total cholesterol and HDL and LDL cholesterol) were obtained early in the morning. For research purposes additional blood was taken at these evaluation moments. In a subset of patients dietary intake was assessed using a self-administered 3-day food diary, which was checked by a dietitian, and subsequently caloric intake was computed. Also an estimate of the patients activity, expressed as ‘metabolic equivalent of task’ (MET) minutes per week, *via* the International Physical Activity Questionnaire was assessed.

Similarly, in a subset of patients DEXA-scans were available, which were performed using either the Lunar Prodigy Advance or the Lunar iDEXA [both: GE Healthcare, Madison, WI, USA. Measurements are comparable between these scans (28)]. We assessed total mass, fat mass (kg), fat-free mass (kg).

### Monocyte immunophenotyping and serum sCD163 measurement

Monocyte immunophenotypes were analyzed in freshly obtained whole blood samples by flow cytometry using a FACS Canto II (Becton Dickinson) in the diagnostic facility of the Laboratory Medical Immunology under strict quality guidelines (ISO15189) (29). The following phenotypical markers (antibody clones and fluorescent labels mentioned in parentheses) were used to identify monocyte subsets and inflammatory activation status: CD45 (HI30-PO, Invitrogen), CD14 (MO-P9-APC-H7, BD Biosciences), CD16 (3G8-PE-Cy7, BD), CD64 (10.1-PerCP-Cy5.5, BD), HLA-DR (L243-PB, Biolegend), CD300e/IREM2 (UP-H2-APC, Immunostep) and CD36 (CLB-IVC7-FITC, Sanquin). An overview of these markers, their expression and function is provided in **Supplementary table 1**. Flowcytometry data were analyzed using Infinicyt software (Cytognos, Salamanca, Spain) and median fluorescence intensities (MFI) were determined. Monocytes were identified by being CD45 positive, their medium-low position on forward scattering plot and their medium-low position on the sideway scattering plot, Subsequently, classical (CD14++CD16-), intermediate (CD14++CD16+) and non-classical monocytes (CD14+16++) were identified. A detailed overview of the gating strategy is given in **Supplementary data 2**. Serum sCD163, as marker for tissue macrophage activity, was determined by ELISA as previously described (7).

### Statistics

SPSS version 25 was used for statistical analysis and Prism Graphpad version 8 for the visual representation of the data. Normally distributed data in tables are presented as mean + standard deviation (SD), non-normally distributed data as median + range. Differences in monocyte characteristics between specific patient subgroups (e.g. differing in age, sex) were assessed by an independent T-test for parametric data, and otherwise a Mann-Whitney-U test. Baseline monocyte counts were correlated to clinical characteristics using a Pearson or Spearman correlation whenever appropriate.

To detect changes throughout the intervention, we used repeated measurements ANOVA with *post-hoc* T-test or Friedmans test with a *post-hoc* Wilcoxon rank test. We calculated changes in monocyte markers by the following formula: ((MFI value new - MFI value old)/MFI old) X 100%. Changes in monocyte subset distribution and monocyte markers were correlated to changes in BMI and waist circumference (WC) using a Pearson or Spearman correlation. Overall, a p-value < 0.05 was considered statistically significant.

## Results

### Baseline clinical and monocyte characteristics

In total, 73 patients with obesity were included (78.1% female, mean age 43.1 (± 11.3) years). Mean BMI was 37.6 kg/m^2^ ( ± 5.6), mean waist circumference was 105.8 cm (± 15.3) and 35 patients fulfilled the criteria of the metabolic syndrome (MetS) (47.9%). Males had significantly higher weight, waist circumference (WC) and blood glucose levels than females (p<0.001, p<0.001, p=0.036 respectively). Additional clinical baseline characteristics are provided in **Table 1**.

**Table 1:** Baseline characteristics in patients with obesity.

	Female	Male	Significance Female/Male
N	57	16	
Age, years (Mean, SD)	42.40 (11.45)	45.69 (10.64)	0.307
Height, cm (Mean, SD)	1.67 (0.07)	1.80 (0.04)	<0.001
Weight, kg (Mean, SD)	104.72 (17.45)	122.12 (11.46)	<0.001
BMI, kg/m2 (Mean, SD)	37.59 (5.96)	37.67 (3.99)	0.373
Waist circumference, cm (Mean, SD)	102.59 (14.56)	117.91 (12.12)	<0.001
Fasting blood glucose level, mmol/L (Mean, SD)	5.58 (1.39)	6.50 (1.69)	0.036
HOMA-IR (Mean, SD)	5.75 (6.07)	8.89 (6.31)	0.091
HDL cholesterol level (Mean, SD)	1.38 (0.33)	1.09 (0.23)	0.001
Triglyceride levels (Mean, SD)	1.42 (0.83)	2.14 (1.34)	0.010
MetS present (N) (%)	26 (54.2)	9 (56.2)	1.000
Participants in protocol E-health (N) (%)	28 (49.1)	6 (37.5)	0.589

SD standard deviation, MetS Metabolic syndrome [definition based on Alberti et al (25)].

In our patient group, average cell counts were 4.1 x 10^5^/ml for the total monocyte population, 31.3 x 10^4^/ml for CM, 0.91 x 10^4^/ml for IM and 3.6 x 10^4^/ml for NCM (**Table 2**). Mean relative monocyte subset distributions were: CM = 86.9%, IM = 2.6% and NCM = 10.5%. We observed no significant differences in absolute monocyte counts between the two sexes, between patients with or without MetS and between age categories (<50 and ≥50 years, **Supplementary data 3**). Regarding monocyte frequencies: older patients (≥50 years) had a lower percentage of CM and higher percentage of NCM (p=0.01 and p=0.02 respectively, **Supplementary data 3A**). With respect to marker expression, also only limited differences were found between subgroups: HLA-DR expression by IM was higher in older individuals (p=0.04, **Supplementary Data 3A**), and CD300e expression by CM and NCM was significantly increased in subjects with MetS (p=0.02 and 0.03 respectively, **Supplementary data 3B**).

**Table 2:** Leukocyte subset counts and monocyte subset phenotypes at baseline of individuals with obesity.

		Female	Male	p-value
			
N		57	16	
Total leukocyte count (10E6/ml), (mean (SD))		6.8 (1.4)	7.0 (2.0)	0.72
Total neutrophil count (10E6/ml), (mean (SD))		3.9 (1.2)	4.3 (1.8)	0.28
Total lymphocyte count (10E6/ml), (mean (SD))		2.2 (0.7)	1.9 (0.5)	0.12
Total eosinophil count (10E6ml), (mean (SD))		0.3 (0.6)	0.3 (0.2)	0.94
Total monocyte count (10E6ml), (mean (SD))		0.3 (0.1)	0.4 (0.1)	0.23
Relative CM (%) (mean (SD))		87.0 (4.0)	86.6 (3.1)	0.73
*Relative IM (%) (median [IQR])*		*2.4 [1.9, 3.0]*	*2.4 [1.9, 2.8]*	*0.57*
Relative NCM (%) (mean (SD))		10.3 (3.8)	10.9 (3.0)	0.55
Absolute number of CM (10E4/ml), (mean (SD))		29.9 (10.6)	33.2 (11.4)	0.28
Absolute number of IM (10E4/ml), (mean (SD))		0.9 (0.4)	0.9 (0.5)	0.79
Absolute number of NCM (10E4/ml), (mean (SD))		3.4 (1.4)	4.1 (1.6)	0.07
MFI CD36	CM (10E3), (mean (SD))	60.1 (24.7)	57.9 (22.4)	0.75
	IM (10E3), (mean (SD))	65.5 (31.1)	70.5 (25.0)	0.56
	NCM (10E3), (mean (SD))	27.3 (20.2)	27.9 (16.7)	0.91
MFI CD64	CM (10E3), (mean (SD))	9.9 (3.5)	9.1 (2.6)	0.42
	IM (10E3), (mean (SD))	7.6 (3.5)	7.0 (2.4)	0.54
	*NCM (10E3) (median [IQR])*	*1.5 [0.9, 2.4]*	*1.5 [1.0. 2.6]*	*0.73*
MFI CD14	CM (10E3), (mean (SD))	20.8 (4.8)	19.8 (3.0)	0.44
	IM (10E3), (mean (SD))	16.3 (5.0)	15.0 (3.3)	0.34
	NCM (10E3), (mean (SD))	2.6 (1.4)	2.2 (0.9)	0.3
MFI CD16	CM (10E3), (mean (SD))	1.9 (0.7)	1.7 (0.5)	0.41
	IM (10E3), (mean (SD))	15.9 (6.1)	15.7 (6.4)	0.89
	NCM (10E3), (mean (SD))	27.3 (10.8)	26.8 (11.0)	0.86
MFI CD45	CM (10E3), (mean (SD))	6.5 (1.2)	6.4 (0.9)	0.74
	IM (10E3), (mean (SD))	8.5 (1.2)	8.7 (1.0)	0.48
	NCM (10E3), (mean (SD))	7.4 (1.1)	7.7 (0.8)	0.32
MFI HLA-DR	CM (10E3), (mean (SD))	6.5 (2.9)	6.0 (2.9)	0.57
	IM (10E3), (mean (SD))	22.6 (8.7)	21.0 (8.1)	0.52
	NCM (10E3), (mean (SD))	10.6 (4.2)	8.9 (3.1)	0.14
MFI CD300e	*CM (10E3) (median [IQR])*	*3.4 [2.5, 3.8]*	*3.5 [2.9, 5.0]*	*0.55*
	*IM (10E3) (median [IQR])*	*5.2 [4.0. 6.6]*	*5.8 [4.4, 9.4]*	*0.42*
	*NCM (10E3) (median [IQR])*	*3.2 [2.7, 4.4]*	*4.2 [3.0. 5.3]*	*0.21*

Normally distributed data are presented as mean (standard deviation), non-normally distributed data as median + interquartile range and in italic font. Independent T-tests were used to determine differences between normally distributed data, the Mann-Whitney U test for non-normally distributed data. A significance level of 0.05 was used. CM classical monocytes, IM intermediate monocytes, NCM non-classical monocytes, SD standard deviation, IQR interquartile range, MFI median fluorescence intensity.

### Monocyte characteristics are associated with parameters of obesity and systemic inflammation

Investigating baseline characteristics, we found no significant associations between absolute or relative monocyte counts and BMI (**Table 3**; **Supplementary Figure 4A**). The IM subset frequency was weakly negatively associated to WC (rho=-0.234, p=0.048, **Table 3**; **Supplementary Data 4B**). There were no other correlations between absolute or relative monocyte counts and WC.

**Table 3:** Correlation between monocyte characteristics and relevant obesity parameters at baseline.

	Parameter	BMI	WC	sCD163	HOMA-IR	HDL-cholesterol	Triglycerides
Absolute monocyte count	Total	0.052	0.189	0.259*	-0.032	-0.220	0.197
	CM	0.050	0.178	0.243	-0.031	-0.236*	0.185
	IM	0.053	0.032	0.420***	-0.049	-0.033	-0.042
	NCM	0.044	0.217	0.177	-0.020	-0.038	0.257*
Relative monocyte count	CM	-0.071	-0.059	-0.027	0.048	-0.237*	-0.049
	*IM*	*-0.139*	-0.234*	*0.195*	*-0.026*	*0.188*	*-0.188*
	NCM	0.063	0.093	-0.046	-0.038	0.203	0.106
CD36	CM	-0.204	-0.169	-0.003	0.042	-0.109	0.135
	IM	-0.262*	-0.176	0.033	-0.027	-0.138	0.122
	NCM	-0.262*	-0.173	0.046	-0.086	-0.105	0.135
CD14	CM	-0.283*	-0.314**	0.099	0.066	-0.100	0.202
	IM	-0.301**	-0.279*	0.067	0.023	-0.103	0.145
	NCM	-0.217	-0.200	0.046	0.022	0.021	0.012
CD16	CM	-0.149	0.003	0.076	0.125	-0.148	0.138
	IM	0.015	0.136	-0.058	0.296*	-0.066	0.110
	NCM	-0.123	0.004	-0.005	0.246*	-0.041	-0.018
CD64	CM	-0.118	-0.090	0.017	0.048	0.019	-0.059
	IM	-0.153	-0.088	0.060	0.061	-0.028	-0.026
	*NCM*	*-0.096*	*0.107*	*0.009*	0.252*	*-0.167*	*0.034*
CD45	CM	-0.280*	-0.104	-0.088	0.002	-0.125	0.071
	IM	-0.146	0.117	-0.064	0.101	-0.179	0.309**
	NCM	-0.189	0.058	0.028	0.038	-0.091	0.338**
HLA-DR	CM	-0.279*	-0.173	0.007	-0.223	0.117	-0.014
	IM	-0.005	0.014	-0.008	-0.184	0.098	0.084
	NCM	-0.136	-0.165	0.176	-0.232	0.103	0.057
CD300e	*CM*	0.268*	0.295*	*0.115*	*0.145*	*-0.155*	0.246*
	*IM*	0.233*	0.311**	*0.100*	*0.136*	*-0.070*	0.299*
	*NCM*	*0.175*	0.286*	*0.144*	*0.131*	*-0.122*	0.327**
sCD163		0.166	0.165	NA	0.065	-0.156	0.021

*: p<0.05

**: p<0.01

***: p<0.001

Pearson correlation coefficients are shown between the mean fluorescent index (MFI) of relevant phenotypical markers of monocytes. Correlation coefficients in Italic are calculated using Spearman’s correlation coefficient. Correlation coefficients in **bold** are significant at p < 0.05. NA, not applicable; CM, classical monocytes; IM, intermediate monocytes; NCM, non-classical monocytes; BMI, body mass index; WC, waist circumference; HDL, cholesterol high density lipoprotein cholesterol.

Interestingly, in our patients with obesity, we observed a significant association between monocyte counts and signs of systemic inflammatory activation, as the absolute number of total monocytes and the absolute count of IM correlated positively to circulating sCD163 levels (r=0.420, p<0.001, **Table 3**; **Supplementary Figure 4C**), and absolute CM counts had a positive trend to sCD163 levels (r=0.243, p=0.053).

Regarding monocyte inflammatory marker expression, we found several significant associations with obesity parameters. CD14 expression by CM and IM correlated negatively with WC and BMI (**Table 3**; **Supplementary Figure 4D**, all p<0.05). In contrast, CD300e expression on all monocyte subsets correlated positively to WC and BMI (**Table 3**; **Supplementary Figure 4E**, all p<0.05, except for CD300e in NCM versus BMI, which was not significant). CD36 expression by IM and NCM correlated negatively with BMI (**Table 3**, **Supplementary Figure 4F**). CD45 expression by CM negatively correlated with BMI, as did HLA-DR expression on CM (**Table 3**, **Supplementary Figure 4G, H**). Monocytic CD16 and CD64 expression showed no significant correlations with WC or BMI (data not shown).

For metabolic parameters, we saw a different profile (**Table 3** and **Supplementary Data 4I**). HOMA-IR correlated positively to CD16 expression by IM and NCM (r=0.296 and 0.246 respectively, both p<0.05), as well as CD64 expression by NCM (rho=0.252, p<0.05). HDL-cholesterol levels only correlated negatively to the absolute and relative amount of classical monocytes (r=-0.236 and -0.237 respectively, both p<0.05). Triglyceride levels had a positive association to the absolute number of NCM (r=0.257, p<0.05), CD45 expression by IM and NCM (r=0.309 and r=0.338, p<0.01) and with CD300e expression by CM, IM and NCM (rho=0.246, 0.299 and 0.327, all p<0.05).

### Changes in monocyte parameters during a combined lifestyle intervention program

From all patients who started the CLI program, complete data at baseline and T1 (10 weeks) could be obtained from 51 participants, while for T2 (1.5 years) complete data were available from 35 participants. Reasons for loss-to-follow-up were: pregnancy (n=2), not showing up at evaluation moments (n=2), difficulty in completing the program (n=1), and no specified reason (n=11).

The effects of CLI on anthropometric parameters are summarized in **Table 4**. In the first 10 weeks, participants (n=51) achieved significant decreases in weight and WC, and lost a significant amount of fat mass. After 1.5 years (n=35), weight loss remained stable and WC and fat mass increased slightly, but was still smaller than baseline. Participants who completed the full program did not differ regarding baseline characteristics or T1 characteristics from those who dropped out. In the **Supplementary Data 5**, we show the mean self-reported daily caloric intake, as assessed from nutritional diaries, as well as mean MET-minutes of whom this was available.

**Table 4:** Clinical parameters before, during and after a combined lifestyle intervention.

	Clinical parameter	T0	T1	T2
Participants with complete data at baseline and first evaluation (n=51)	Weight (Kg)	107.7	103.0 ^a^	n/a
	BMI (Kg/m^2^)	37.2	35.6 ^a^	n/a
	Waist circumference (cm)	103.9	97.8 ^a^	n/a
	DXA fat mass (kg) ^d^	49.2	46.5 ^a^	n/a
	DXA fat free mass (kg) ^d^	54.0	53.4	n/a
	Fasting glucose	5.7	5.4 ^a^	n/a
	HDL	1.4	1.3	n/a
	Triglycerides	1.4	1.2 ^a^	n/a
Subgroup of participants with complete data at baseline, first and second assessment (n=35)	Weight (kg)	108.4	103.2 ^a^	103.9 ^ab^
	BMI (kg/m^2^)	37.4	35.6 ^a^	35.8 ^ab^
	Waist circumference (cm) ^c^	103.2	97.7 ^a^	102.8 ^ab^
	DXA fat mass (kg) ^d^	49.2	45.4 ^a^	45.8
	DXA fat free mass (kg) ^d^	54.8	54.1	54.5
	Fasting glucose	5.6	5.5	5.7
	HDL	1.4	1.3	1.4 ^b^
	Triglycerides	1.6	1.3 ^a^	1.6 ^b^

Follow-up clinical characteristics during intervention. All data are normally distributed and are presented as mean + SD. Paired T-tests and a significance level of 0.05 was used.

a: significantly different from T0 (p < 0.05).

b: significantly different form T1 (p < 0.05).

c: complete waist circumference data in n=23 participants.

d: complete data on DEXA-scans available in n=14 participants (at T0 and T1) and n=12 (at T0, T1 and T2).

BM, body mass index (kg/m^2^). n/a, not applicable.

**Figure 1:**
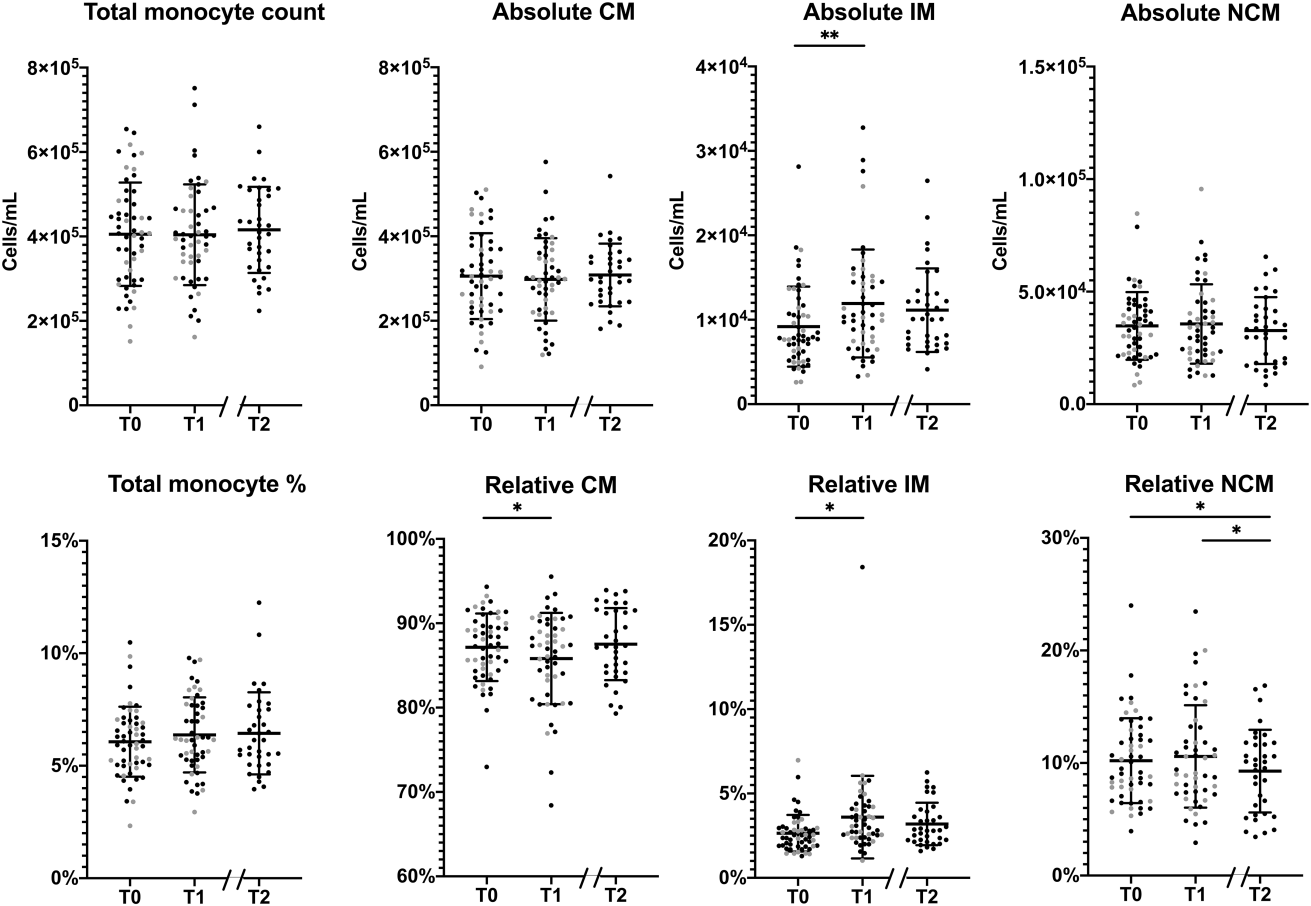
Changes in the monocyte subset distribution during the lifestyle intervention. Changes in absolute (upper row) and relative (lower row) monocyte levels in total monocytes, classical monocytes (CM), intermediate monocytes (IM) and non-classical monocytes (NCM) between T0 and T1 of patients who had a complete follow-up at T1 (n=51) and between T0 and T2 patients who completed the program (n=35). Individuals who had follow-up until T1 are represented as grey dots, individuals with follow-up until T2 are represented as black dots. Normally distributed data, (total monocyte counts, absolute CM/NCM and relative CM/NCM), are presented as mean ± SD. Non-normally distributed data, (absolute and relative IM), are presented as median and range. Paired T-tests were used to determine the differences between T0 and T1, and between T0 and T2 for normally distributed data, the Wilcoxon signed ranks test for non-normally distributed data. *p < 0.05, **p < 0.01.

Considering monocyte parameters, we observed that the absolute total monocyte count did not change significantly during the program (**Figure 1**). For the subset distribution, in the 51 patients who completed the first part of the program a significant increase in absolute IM counts was observed (p=0.039), corresponding to an average relative increase of IM from 2.7% at baseline to 3.6% at T1 (p=0.008). Meanwhile, relative CM levels at baseline were 87.1%, which decreased to 85.8% at T1 (p=0.025), while relative NCM remained stable at T1. Among the 35 patients who completed the full, 1.5 year program, no other significant changes were observed between T1 and T2 for absolute or relative counts of monocyte subsets, except for a decrease in the relative amount of NCM (10.8% at T1 and 9.2% at T2; p=0.043). Remarkably, changes in anthropometric parameters between T0 and T1 or between T0 and T2 were not associated to changes in monocyte counts.

**Figure 2:**
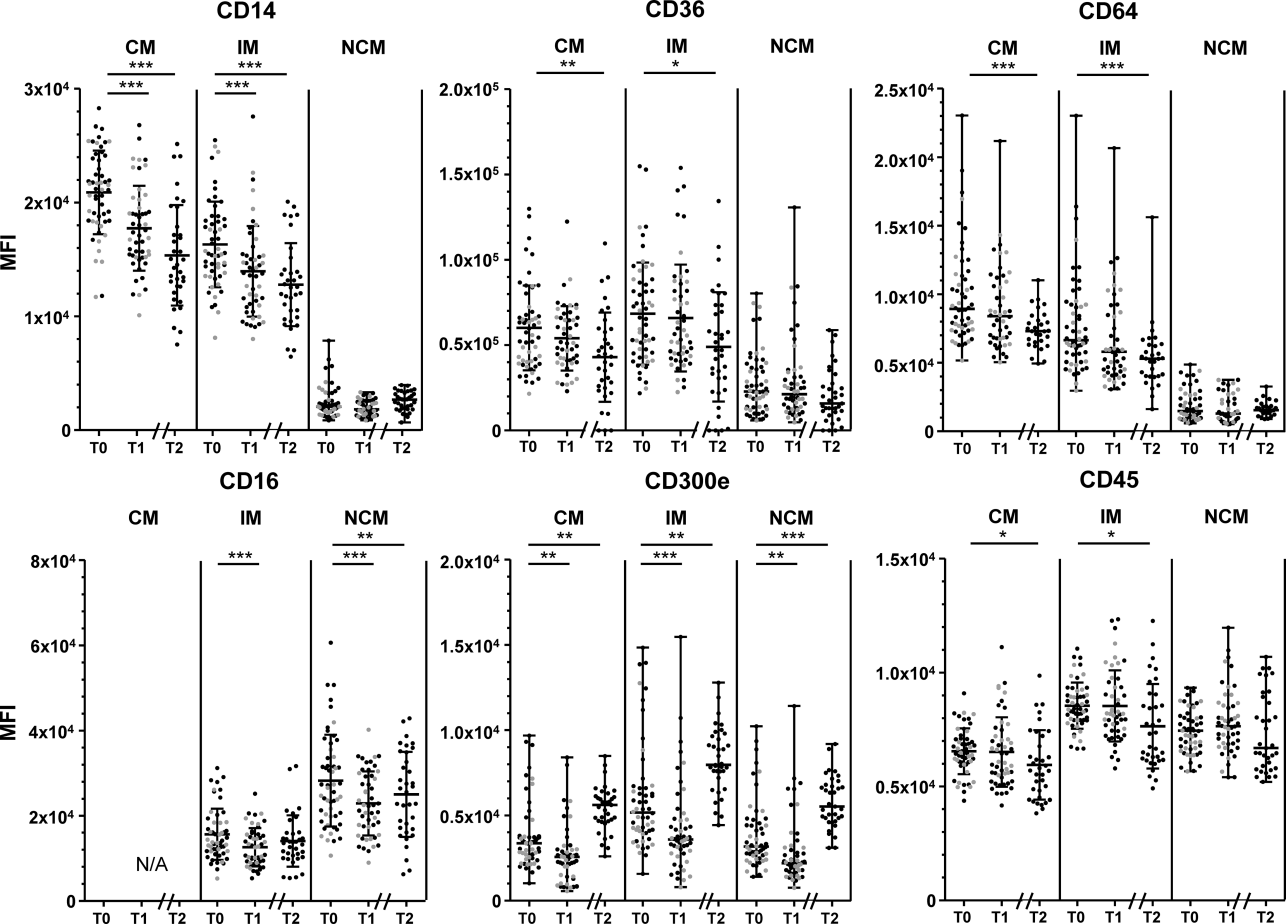
Changes in marker expression during the lifestyle intervention. Changes in CD14 expression (upper left), CD36 (upper middle) CD64 expression (upper right), CD16 (lower left), CD300e (lower middle) and CD45 (lower right), in classical monocytes (CM), intermediate monocytes (IM) and non-classical monocytes (NCM) between T0 and T1 of patients who had a complete follow-up at 10 weeks (T1, n=51) and at 1.5 years (T2, n=35). Individuals who had follow-up until T1 are represented as grey dots, individuals with follow-up until T2 are represented as black dots. CD14 expression by CM and IM, CD36 expression by CM and IM, CD16 expression by all subsets, and CD45 expression by CM and IM are normally distributed and are presented as mean + SD. CD300e and CD64 expression by all subsets, CD14 expression, CD36 expression and CD45 by NCM are presented as median + range. Paired T-tests were used to determine the differences between T0 and T1, and between T0 and T2 for normally distributed data, the Wilcoxon signed ranks test for non-normally distributed data. Differences between T0 and T1 were calculated in n=51 participants, differences between T1-T2 and T0-T2 were calculated in n=35 participants. N/A: not applicable. MFI: median fluorescence intensity. *p < 0.05, **p < 0.01, ***p < 0.001.

Changes in monocyte marker expression throughout the intervention are shown in **Figure 2**. CD14 expression by CM and IM decreased significantly between T0 and T1, and showed a further decrease at T2 (all p<0.001). CD16 expression by IM and NCM decreased between T0 and T1 (both p<0.001), and stabilized at T2. CD300e expression decreased slightly in all monocyte subsets between T0 and T1 (all p<0.01), but showed a strong increase towards T2 (all p<0.01). CD64, CD36 and CD45 expression by CM and IM decreased, reaching statistical significance for T2 (p<0.001 for CD64, all p<0.05 for CD36 and CD45). HLA-DR expression did not change on any of the measured monocyte subsets throughout the intervention period (**Supplementary Figure 6**).

In general, changes in CD14, CD36, CD64, CD45 and CD300e were not associated with changes in BMI or WC. Only the initial decrease in WC between baseline and T1 correlated to a decrease in CD14 expression by IM (r= 0.318, p=0.026; **Supplementary Figure 7**), whereas at T2 the decrease in WC correlated to an increase in CD14 expression (r= -0.415, p=0.049, **Supplementary Figure 7**). There were however no associations between changes in CD14 expression of IM and BMI, nor associations between WC and CD14 expression by CM (p>0.1).

## Discussion

In the current study, we investigated peripheral blood counts of the three main monocyte subsets as well as specific surface marker expression levels by these monocyte subsets in individuals with obesity. We studied associations between monocyte subset composition and their phenotype and parameters of obesity, as well as their respective changes upon a lifestyle intervention.

With respect to the absolute and relative numbers of distinct monocyte subsets, we found that these were not correlated at baseline to BMI and WC, except for the frequency of IM, which correlated modestly negatively to WC. We observed that after 1.5 year of lifestyle modifications, leading to significant but modest decreases in weight and waist circumference, there was a subtle increase in absolute and relative numbers of IM in the initial phase of the program. However, at the end of the program this increase was not maintained, and the change in IM lost statistical significance compared to baseline. Furthermore, we saw a small but significant decrease in the relative number of NCM at the end of the program, compared to both baseline and T1.

Previously, others have investigated the relations between monocyte subset counts and parameters of obesity. Most of these studies reported that increasing counts of CD16+ monocytes (IM and NCM) are associated to increasing severity of obesity (14, 15, 30) or with components of MetS (3, 20, 31). In our population, the only significant association of monocyte counts with obesity parameters at baseline was the negative association between the relative number of IM with waist circumference, which seems contradictory to the earlier findings. As most other correlation coefficients were positive, though non-significant, this could indicate a power problem. This seems to be a more general issue in this field of studies with extensive immunological measurements, as many previously published studies had a comparable or sometimes even smaller number of participants compared to ours (16, 20–22, 30). Importantly, other cohorts often included lean or overweight individuals (15), or individuals with more severe obesity (14), which may limit the comparability as our cohort showed less dispersion in BMI.

Interestingly, we found strong associations between the total number of monocytes and sCD163, and between the number of IM and this indicator of systemic macrophage activation. We previously found sCD163 to be increased in this cohort of patients with obesity, and particularly in those who also have the MetS (7). The association between total monocytes, and particularly IM, and sCD163 can be interpreted as indication of a generalized, systemic activation of at least the monocyte/macrophage arm of the innate immune system that is present in individuals with obesity. This is possibly linked *via* the chemokine CCL2, which is increased in obesity (7) and important in monocyte mobilization and correlated to the number of monocytes in circulation (32).

After the CLI, we observed no major changes in the total monocyte and subset counts. Absolute and relative IM counts subtly increased after the first ten weeks, but these changes were not persistent at the end of the program, whereas relative NCM numbers slightly decreased only at the end of the program, and absolute NCM counts were stable. Altogether, our findings are similar to what has been published before for short-term lifestyle interventions, describing minimal or inconsistent changes in monocyte subpopulations in response to short-term diets or lifestyle changes (14, 20–22, 33). However, our finding that IM counts significantly increase after ten weeks of CLI is not observed in other studies. An explanation for this remains speculative, but might be related to the essentially different approach in our program, characterized by the healthy diet, combined with exercise and behavioral adaptation in a prolonged trajectory. Hence, despite the unique insights on the longer term that our data provide, we found insufficient evidence for consistent changes in monocyte subset distribution following lifestyle changes in individuals with obesity.

In contrast to the lack of consistent associations of the quantity and frequency of monocytes and subpopulations to obesity parameters, and their rather limited changes in response to the CLI, we found that the phenotypical characteristics of the monocytes had stronger baseline associations and more pronounced changes after the intervention. At baseline, patients who had higher BMI and WC had lower CD14 expression by CM and IM, and higher CD300e expression by all subsets. During the intervention, we found that CD14 expression by CM and IM, decreased in the first ten weeks and kept decreasing at follow-up. Also, CD36, CD45 and CD64 expression by CM and IM decreased over the full program, as well as CD16 expression by NCM. The highly significant, continuing decrease of CD14 expression is surprising, and seemingly inconsistent with our finding that CD14 expression correlates negatively to BMI and waist circumference, as both BMI and waist circumference diminished during the intervention. These contradictory findings suggest that the immunological changes that take place in obesity are complex and may not simply reverse after weight loss.

Concerning the expression of monocyte activation marker CD300e, we found that its expression by all subsets decreased in the first ten weeks of the program. Since we also found that CD300e positively correlated to BMI and WC, it is consistent with our finding that CD300e decreased when patients initially lost weight. Puzzlingly, while patients maintained weight loss during the second leg of the program, CD300e expression by all monocyte subsets increased, resulting in a significantly higher expression level at T2 than T0. Of note, results of the marker expression assessment were not affected by inter-run variability as instrument settings were standardized throughout, and inclusion of patients ran over a significant period of time causing overlap between determinations of different time points in different patients, making a technical explanation for these surprising findings highly unlikely.

To our knowledge, our study is the first to describe CD300e expression of monocytes in obesity, and the response to an intervention. It can be speculated that its increased expression with increasing obesity is indicative of the pro-inflammatory status in obesity, and that it thus decreases after initial weight loss. In the subsequent weight maintenance phase CD300e expression in all subsets increases again sharply, which may indicate again that a different inflammatory response state is achieved.

To summarize changes in monocyte surface markers upon CLI, we found a general pattern of downregulation of cell surface markers CD14, CD16, CD36, CD45 and CD64 on relevant subsets during the program, with the remarkable exception of increased CD300e in the second leg. Since monocyte cell surface markers are indicative of their inflammatory and activation status, the decreased expression in activating receptors causes monocytes to be less responsive upon encountering appropriate triggers. This suggests that monocytes’ contribution to the chronic, systemic inflammation characterizing obesity is lowered or altered. This may be related to changes in T-cell homeostasis that we described earlier in this same cohort (7), where we found less T-cell activation and increased regulatory T-cells after the lifestyle intervention. Importantly, the inflammatory mark-up that is seen after lifestyle changes, as indicated by changes in innate and adaptive immune cell profile (7) and levels of inflammatory cytokine and other mediators such as sCD163, differs from mere “reversal” of the obesity, and may also be subject to changes over time. Together, this indicates the development of a novel inflammatory “post-weight-loss” setpoint, that is different from the phenotype that is seen in lean individuals or individuals with less severe obesity who are not yet treated. This phenomenon may be part of the general memory of the innate immune system. Such an altered immunological phenotype in obesity likely translates into modified responses to environmental triggers, which is known as ‘trained immunity’ (34). Mechanistically, this is thought to be induced by epigenetic changes mediated by chronic metabolic challenges.

In our cohort, changes in monocyte characteristics during CLI were not directly related to changes in BMI and WC. This suggests that distinct mechanisms operate in the regulation of metabolic and immune parameters related to obesity. We have previously shown that the beneficial effects of this lifestyle intervention extend beyond weight loss alone, and include also metabolic and psychological improvements (24). Perhaps some of the changes we observed at monocyte level can be related to improvements in mental status or body composition, such as fat mass or fat-free mass (of which we had too little data to perform valid statistical correlations), instead of weight loss alone.

## Strengths and limitations

Strengths of this research are the extensive immunological phenotyping that extends beyond monocyte counts but also includes activation markers. Also the multidisciplinary approach of this intervention may be considered a strong point. Many lifestyle interventions in the treatment of obesity focus solely on diet or exercise, whereas our intervention combines a healthy diet with exercise and elements of behavioral therapy (24). Moreover, patients were followed for 1.5 years, while previous studies that investigate monocytes focus on short term outcomes, thereby ignoring the weight regain that frequently occurs after short-term weight loss.

On the other hand, our work also has important limitations. During this long follow-up there was a substantial number of participants that dropped out of the program or of whom we did not have complete data at the second evaluation. As for many participants it is not known why they dropped out of the program, this may have induced a selection bias. Next, our sample size is relatively small, which limits statistical power and hampered us to perform valid subgroup analyses, such as sex-differences. Next, data regarding adherence to the lifestyle-intervention are lacking or may be subject to recall-bias, which is a known phenomenon for studies involving lifestyle interventions (35). Another weakness of the study is that we could not include a control cohort of individuals without obesity, nor follow individuals with obesity that were not treated, which would have enabled a direct comparison between these groups.

Next, for future research it would be highly interesting to include functional studies of monocytes, for instance the response to an inflammatory stimulus such as lipopolysaccharides (LPS), or include gene expression profiles of the monocyte subsets, to obtain a more extensive view on the inflammatory profile of the monocytes.

## Final remarks

In conclusion, we found that patients with obesity who followed a lifestyle intervention showed a persistent, long-term reduction of several monocyte surface activation markers, despite only modest changes in the counts of monocyte subpopulations. Our findings suggest that in obesity, the inflammatory status of monocytes may be beneficially affected towards a novel, different phenotype after a comprehensive lifestyle intervention.

## Data availability statement

The datasets presented in this article are not readily available because data sharing is limited as the combination of specific characteristics may lead to identification of our subjects, this will be decided upon request. Requests to access the datasets should be directed to e.vanrossum@erasmusmc.nl.

## Ethics statement

The studies involving human participants were reviewed and approved by Medical Ethical committee of the Erasmus MC. The patients/participants provided their written informed consent to participate in this study.

## Author contributions

EV: conceptualization, data analysis, writing DM: data-analysis, writing TK: conceptualization, resources, writing NN: conceptualization, resources, data analysis, writing ER: conceptualization, funding acquisition, resources, methodology, supervision, writing WD: conceptualization, resources, data-analysis, methodology, supervision, writing PL: conceptualization, resources, data-analysis, methodology, supervision, writing. All authors contributed to the article and approved the submitted version.

## Funding

ER was supported by the Elisabeth Foundation, a non-profit organization supporting academic obesity research; Netherlands Organization of Scientific Research NWO; ZonMW Vidi, Grant/Award Number: 91716453.

## Acknowledgments

The authors would like to thank Renate Meeusen for her valuable contribution as a data manager, facilitating the current study. Lastly, we would like to thank all patients for their willingness to participate in the current study.

## Conflict of interest

The authors declare that the research was conducted in the absence of any commercial or financial relationships that could be construed as a potential conflict of interest.

## Publisher’s note

All claims expressed in this article are solely those of the authors and do not necessarily represent those of their affiliated organizations, or those of the publisher, the editors and the reviewers. Any product that may be evaluated in this article, or claim that may be made by its manufacturer, is not guaranteed or endorsed by the publisher.

## References

[1] WHO. Factsheet obesity and overweight. (2021). Available at: https://www.who.int/news-room/fact-sheets/detail/obesity-and-overweight.

[2] Phillips CL Grayson BE. The immune remodel: Weight loss-mediated inflammatory changes to obesity. Exp Biol Med (Maywood) (2020) 245(2):109–21. doi: 10.1177/1535370219900185 PMC7016415 31955604

[3] Friedrich K Sommer M Strobel S Thrum S Bluher M Wagner U. Perturbation of the monocyte compartment in human obesity. Front Immunol (2019) 10:1874. doi: 10.3389/fimmu.2019.01874 31440251 PMC6694869

[4] Chakarov S Bleriot C Ginhoux F. Role of adipose tissue macrophages in obesity-related disorders. J Exp Med (2022) 219(7):e20211948. doi: 10.1084/jem.20211948 35543703 PMC9098652

[5] Ni Y Ni L Zhuge F Xu L Fu Z Ota T. Adipose tissue macrophage phenotypes and characteristics: The key to insulin resistance in obesity and metabolic disorders. Obes (Silver Spring) (2020) 28(2):225–34. doi: 10.1002/oby.22674 31903735

[6] Zatterale F Longo M Naderi J Raciti GA Desiderio A Miele C. Chronic adipose tissue inflammation linking obesity to insulin resistance and type 2 diabetes. Front Physiol (2019) 10:1607. doi: 10.3389/fphys.2019.01607 32063863 PMC7000657

[7] van der Zalm IJB van der Valk ES Wester VL Nagtzaam NMA van Rossum EFC Leenen PJM. Obesity-associated t-cell and macrophage activation improve partly after a lifestyle intervention. Int J Obes (Lond) (2020) 44(9):1838–50. doi: 10.1038/s41366-020-0615-6 32678324

[8] Fjeldborg K Christiansen T Bennetzen M JM H SB P Richelsen B. The macrophage-specific serum marker, soluble cd163, is increased in obesity and reduced after dietary-induced weight loss. Obes (Silver Spring) (2013) 21(12):2437–43. doi: 10.1002/oby.20376 23512476

[9] Zanni MV Burdo TH Makimura H Williams KC Grinspoon SK. Relationship between monocyte/macrophage activation marker soluble cd163 and insulin resistance in obese and normal-weight subjects. Clin Endocrinol (Oxf) (2012) 77(3):385–90. doi: 10.1111/j.1365-2265.2011.04284.x PMC3660104 22098563

[10] Ziegler-Heitbrock L Ancuta P Crowe S Dalod M Grau V Hart DN. Nomenclature of monocytes and dendritic cells in blood. Blood (2010) 116(16):e74–80. doi: 10.1182/blood-2010-02-258558 20628149

[11] Kapellos TS Bonaguro L Gemund I Reusch N Saglam A Hinkley ER. Human monocyte subsets and phenotypes in major chronic inflammatory diseases. Front Immunol (2019) 10:2035. doi: 10.3389/fimmu.2019.02035 31543877 PMC6728754

[12] Patel AA Zhang Y Fullerton JN Boelen L Rongvaux A Maini AA. The fate and lifespan of human monocyte subsets in steady state and systemic inflammation. J Exp Med (2017) 214(7):1913–23. doi: 10.1084/jem.20170355 PMC5502436 28606987

[13] Ivanova EA Orekhov AN. Monocyte activation in immunopathology: Cellular test for development of diagnostics and therapy. J Immunol Res (2016) 2016:4789279. doi: 10.1155/2016/4789279 26885534 PMC4739459

[14] Poitou C Dalmas E Renovato M Benhamo V Hajduch F Abdennour M. Cd14dimcd16+ and cd14+cd16+ monocytes in obesity and during weight loss: Relationships with fat mass and subclinical atherosclerosis. Arterioscler Thromb Vasc Biol (2011) 31(10):2322–30. doi: 10.1161/ATVBAHA.111.230979 21799175

[15] Rogacev KS Ulrich C Blomer L Hornof F Oster K Ziegelin M. Monocyte heterogeneity in obesity and subclinical atherosclerosis. Eur Heart J (2010) 31(3):369–76. doi: 10.1093/eurheartj/ehp308 19687164

[16] Devevre EF Renovato-Martins M Clement K Sautes-Fridman C Cremer I Poitou C. Profiling of the three circulating monocyte subpopulations in human obesity. J Immunol (2015) 194(8):3917–23. doi: 10.4049/jimmunol.1402655 25786686

[17] Ghanim H Aljada A Hofmeyer D Syed T Mohanty P Dandona P. Circulating mononuclear cells in the obese are in a proinflammatory state. Circulation (2004) 110(12):1564–71. doi: 10.1161/01.CIR.0000142055.53122.FA 15364812

[18] Poitou C Perret C Mathieu F Truong V Blum Y Durand H. Bariatric surgery induces disruption in inflammatory signaling pathways mediated by immune cells in adipose tissue: A rna-seq study. PloS One (2015) 10(5):e0125718. doi: 10.1371/journal.pone.0125718 25938420 PMC4418598

[19] Considine RV. Activated monocytes: Yet another link between systemic inflammation and obesity. J Clin Endocrinol Metab (2014) 99(7):2347–9. doi: 10.1210/jc.2014-2095 25003244

[20] Kim JE Lin G Zhou J Mund JA Case J Campbell WW. Weight loss achieved using an energy restriction diet with normal or higher dietary protein decreased the number of cd14(++)cd16(+) proinflammatory monocytes and plasma lipids and lipoproteins in middle-aged, overweight, and obese adults. Nutr Res (2017) 40:75–84. doi: 10.1016/j.nutres.2017.02.007 28473063

[21] Figueroa-Vega N Marin-Aragon CI Lopez-Aguilar I Ibarra-Reynoso L Perez-Luque E Malacara JM. Analysis of the percentages of monocyte subsets and ilc2s, their relationships with metabolic variables and response to hypocaloric restriction in obesity. PloS One (2020) 15(2):e0228637. doi: 10.1371/journal.pone.0228637 32074122 PMC7029876

[22] Markofski MM Flynn MG Carrillo AE Armstrong CL Campbell WW Sedlock DA. Resistance exercise training-induced decrease in circulating inflammatory cd14+cd16+ monocyte percentage without weight loss in older adults. Eur J Appl Physiol (2014) 114(8):1737–48. doi: 10.1007/s00421-014-2902-1 24832193

[23] Jensen MD Ryan DH Apovian CM Ard JD Comuzzie AG Donato KA. 2013 Aha/acc/tos guideline for the management of overweight and obesity in adults: A report of the american college of cardiology/american heart association task force on practice guidelines and the obesity society. Circulation (2014) 129(25 Suppl 2):S102–38. doi: 10.1161/01.cir.0000437739.71477.ee PMC5819889 24222017

[24] Mohseni M Kuckuck S Meeusen R Lengton R van der Valk E Iyer A. Improvements in physiological and psychological status of patients with obesity in response to a combined lifestyle intervention with cognitive behavioral therapy are not necessarily related to successful weight loss. Endocr Abstr (2022) 81:P585. doi: 10.1530/endoabs.81.P585

[25] Alberti KG Eckel RH Grundy SM Zimmet PZ Cleeman JI Donato KA. Harmonizing the metabolic syndrome: A joint interim statement of the international diabetes federation task force on epidemiology and prevention; national heart, lung, and blood institute; american heart association; world heart federation; international atherosclerosis society; and international association for the study of obesity. Circulation (2009) 120(16):1640–5. doi: 10.1161/CIRCULATIONAHA.109.192644 19805654

[26] Gezondheidsraad. Richtlijnen goede voeding, Vol. 2015. (2015). Available at: https://www.gezondheidsraad.nl/documenten/adviezen/2015/11/04/richtlijnen-goede-voeding-2015.

[27] Kouwenhoven-Pasmooij TA Robroek SJ Ling SW van Rosmalen J van Rossum EF Burdorf A. A blended web-based gaming intervention on changes in physical activity for overweight and obese employees: Influence and usage in an experimental pilot study. JMIR Serious Games (2017) 5(2):e6. doi: 10.2196/games.6421 28373157 PMC5394263

[28] Morrison SA Petri RM Hunter HL Raju D Gower B. Comparison of the lunar prodigy and idxa dual-energy x-ray absorptiometers for assessing total and regional body composition. J Clin Densitom (2016) 19(3):290–7. doi: 10.1016/j.jocd.2015.06.003 PMC4721935 26209017

[29] Boeddha NP Kerklaan D Dunbar A van Puffelen E Nagtzaam NMA Vanhorebeek I. Hla-dr expression on monocyte subsets in critically ill children. Pediatr Infect Dis J (2018) 37(10):1034–40. doi: 10.1097/INF.0000000000001990 29570588

[30] Pecht T Haim Y Bashan N Shapiro H Harman-Boehm I Kirshtein B. Circulating blood monocyte subclasses and lipid-laden adipose tissue macrophages in human obesity. PloS One (2016) 11(7):e0159350. doi: 10.1371/journal.pone.0159350 27442250 PMC4956051

[31] de Matos MA Duarte TC Ottone Vde O Sampaio PF Costa KB de Oliveira MF. The effect of insulin resistance and exercise on the percentage of cd16(+) monocyte subset in obese individuals. Cell Biochem Funct (2016) 34(4):209–16. doi: 10.1002/cbf.3178 27027694

[32] Jordan S Tung N Casanova-Acebes M Chang C Cantoni C Zhang D. Dietary intake regulates the circulating inflammatory monocyte pool. Cell (2019) 178(5):1102–14.e17. doi: 10.1016/j.cell.2019.07.050 31442403 PMC7357241

[33] Wadley AJ Roberts MJ Creighton J Thackray AE Stensel DJ Bishop NC. Higher levels of physical activity are associated with reduced tethering and migration of pro-inflammatory monocytes in males with central obesity. Exerc Immunol Rev (2021) 27:54–66. 33965903

[34] Bekkering S Saner C Riksen NP Netea MG Sabin MA Saffery R. Trained immunity: Linking obesity and cardiovascular disease across the life-course? Trends Endocrinol Metab (2020) 31(5):378–89. doi: 10.1016/j.tem.2020.01.008 32305098

[35] Lichtman SW Pisarska K Berman ER Pestone M Dowling H Offenbacher E. Discrepancy between self-reported and actual caloric intake and exercise in obese subjects. N Engl J Med (1992) 327(27):1893–8. doi: 10.1056/NEJM199212313272701 1454084

