# Supplementary material for: Monocyte adaptations in patients with obesity during a 1.5 year lifestyle intervention
Source: Front Immunol. 2022 Nov 17;13:1022361. doi: 10.3389/fimmu.2022.1022361 (PMC9716348; doi:10.3389/fimmu.2022.1022361)
Supplement: Supplementary file 1 [file DataSheet_1.pdf]

Supplementary data

Table 1) Overview monocyte markers used in this study, their expression and function

Marker	Monocyte subset expression (10, 50, 51)			Description
	classical	intermediate	non-classical	
CD14	++	++	–/+	CD14 functions as a co-receptor with Toll-Like receptor 4 (TLR4) for lipopolysaccharide (LPS) (43). Upon cellular activation, monocytes produce inflammatory cytokines and enzymatically split CD14 from the surface, resulting in circulating soluble CD14.
CD16	–	+	++	CD16 is a low-affinity Fc-receptor (FcγRIII), binding immune complexes. Upon receptor stimulation, monocytes activate antibody-dependent cell-mediated cytotoxicity which enables them to kill virus-infected and cancer cells (52).
CD36	+	++	-	CD36 is a class B scavenger receptor and is primarily known for its role in atherosclerosis (53). It detects various circulating ligands, incl. modified LDL, and is involved in the clearance of cell debris.
CD45	+	+	+	CD45 is a cell surface protein tyrosine phosphatase, present on all leukocytes. It negatively regulates monocyte differentiation and inhibits LPS-stimulated cell activation. (44,45,46).
CD64	++	+	+	CD64 a high affinity Fc-receptor (FcγRI), binding IgG monomers. Upon IgG binding to CD64, a potent inflammatory response is triggered, releasing inflammatory cytokines TNF-alfa among others, into circulation (54).
HLA-DR	+/-	++	+	HLA-DR is an MHC class II surface molecule that is involved in antigen presentation to CD4+ T-cells. HLA-DR expression is elevated by acute inflammatory activation, but downregulated upon strong activation such as in sepsis. Also chronic activation, such as after stressful episodes causes HLA-DR downregulation. As such, HLA-DR expression change is a hallmark for an altered immune status (55).
CD300e	+	+	+	CD300e, also known as IREM2, , is an activating receptor that upon stimulation prevents monocyte apoptosis, triggers inflammatory cytokine production and upregulates co-stimulatory molecules (56).

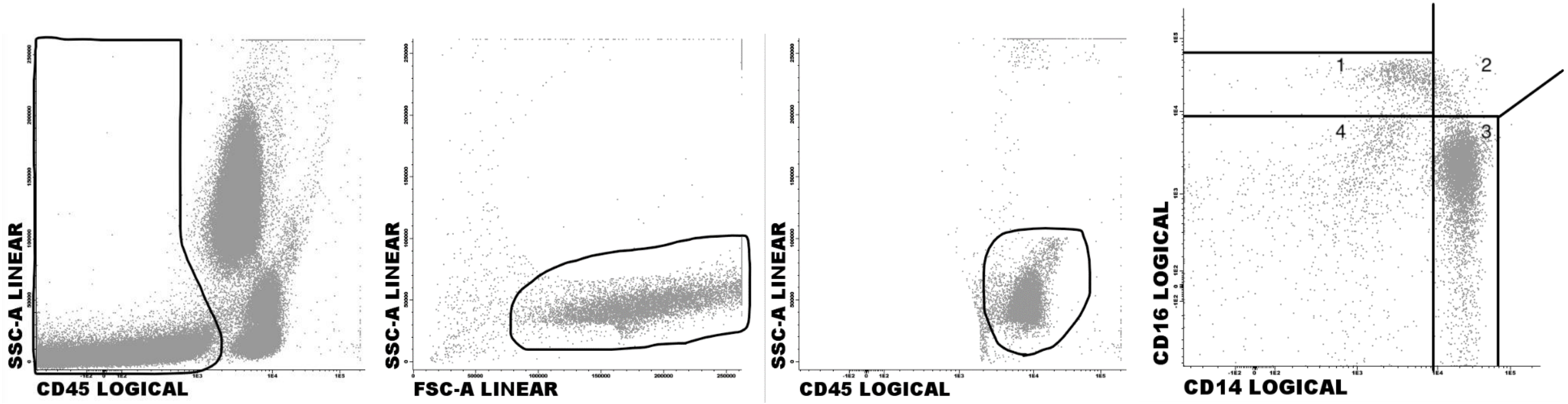

Supplementary data 2:
Gating procedure. A. all CD45 negative events were excluded. B. Lymphocytes and granulocytes were gated-out and monocytes were gated on the basis of forward scatter and sideward scatter. C. Monocyte gating was further refined by gating on CD45 expression and sideward scatter characteristics. .D. Gating strategy to determine non-classical monocytes (1; CD14+/CD16++)), intermediate monocytes (2; CD14++CD16+), classical monocytes (3; CD14++CD16-)) and dendritic cells (4).

Supplementary data 3a. **Monocyte characteristics of individuals with obesity at baseline, stratified by age status.** Normally distributed data are presented as mean (standard deviation), non-normally distributed data as median + interquartile range and in *italic font*. Independent T-tests were used to determine differences between normally distributed data, the Mann-Whitney U test for non-normally distributed data. A significance level of 0.05 was used. *CM* Classical monocytes, *IM* Intermediate monocytes, *NCM* non-classical monocytes, *SD* standard deviation, *IQR* interquartile range, *MFI* Mean fluorescent index.

		**Age <50 years**	**Age ≥50 years**	
n		52	21	**p-value**
Total leukocyte count (10^6/ml), (mean (SD))		**7.1 (1.5)**	**6.1 (1.5)**	**0.01**
Total neutrophil count (10^6/ml), (mean (SD))		**4.2 (1.3)**	**3.4 (1.4)**	**0.03**
Total lymphocyte count (10^6/ml), (mean (SD))		2.2 (0.7)	2.0 (0.6)	0.28
Total eosinophil count (10^6ml), (mean (SD))		0.3 (0.6)	0.3 (0.5)	0.92
Total monocyte count (10^6ml), (mean (SD))		0.4 (0.1)	0.3 (0.1)	0.6
Relative CM (%) (mean (SD))		**87.6 (3.9)**	**85.2 (3.0)**	**0.01**
Relative IM (%) (median [IQR])		2.3 [1.9, 2.9]	2.6 [2.1, 3.6]	0.23
Relative NCM (%) (mean (SD))		**9.8 (3.6)**	**12.0 (3.1)**	**0.02**
Absolute number of CM (10^4/ml), (mean (SD))		31.3 (11.8)	28.9 (7.8)	0.39
Absolute number of IM (10^4/ml), (mean (SD))		0.9 (0.5)	0.9 (0.4)	0.66
Absolute number of NCM (10^4/ml), (mean (SD))		3.4 (1.3)	4.1 (1.7)	0.05
MFI CD36	CM (10^3), (mean (SD))	60.7 (25.1)	56.9 (21.7)	0.55
	IM (10^3), (mean (SD))	67.2 (29.1)	65.1 (32.2)	0.79
	NCM (10^3), (mean (SD))	26.4 (15.7)	30.0 (26.6)	0.48
MFI CD64	CM (10^3), (mean (SD))	9.9 (3.6)	9.2 (2.6)	0.39
	IM (10^3), (mean (SD))	7.6 (3.6)	7.1 (2.4)	0.5
	NCM (10^3) (median [IQR])	1.6 [1.0, 2.5]	1.4 [0.9, 2.1]	0.65
MFI CD14	CM (10^3), (mean (SD))	20.4 (4.4)	21.0 (4.5)	0.56
	IM (10^3), (mean (SD))	15.7 (4.0)	16.9 (6.2)	0.33
	NCM (10^3), (mean (SD))	2.5 (1.2)	2.6 (1.4)	0.77
MFI CD16	CM (10^3), (mean (SD))	1.9 (0.7)	1.8 (0.6)	0.84
	IM (10^3), (mean (SD))	15.5 (6.0)	17.0 (6.4)	0.35
	NCM (10^3), (mean (SD))	26.9 (10.8)	27.9 (10.7)	0.73
MFI CD45	CM (10^3), (mean (SD))	6.5 (1.2)	6.3 (1.2)	0.53
	IM (10^3), (mean (SD))	8.6 (1.2)	8.5 (1.0)	0.83
	NCM (10^3), (mean (SD))	7.4 (1.0)	7.5 (1.0)	0.83
MFI HLA-DR	CM (10^3), (mean (SD))	5.9 (2.8)	7.4 (3.0)	0.05
	IM (10^3), (mean (SD))	**21.0 (8.9)**	**25.5 (6.8)**	**0.04**
	NCM (10^3), (mean (SD))	9.8 (4.1)	11.2 (3.9)	0.2
MFI CD300e	*CM (10^3) (median [IQR])*	3.5 [2.5, 4.8]	3.3 [2.7, 4.8]	0.93
	IM (10^3) (median [IQR])	5.1 [4.0, 7.1]	5.8 [4.3, 7.2]	*0.42*
	NCM (10^3) (median [IQR])	3.4 [2.7, 4.7]	3.4 [2.7, 4.7]	*0.21*

Supplemental data 3b. **Monocyte characteristics of individuals with obesity at baseline, stratified by presence of the metabolic syndrome.** Normally distributed data are presented as mean (standard deviation), non-normally distributed data as median + interquartile range and in *italic font*. Independent T-tests were used to determine differences between normally distributed data, the Mann-Whitney U test for non-normally distributed data. A significance level of 0.05 was used. *CM* Classical monocytes, *IM* Intermediate monocytes, *NCM* non-classical monocytes, *SD* standard deviation, *IQR* interquartile range, *MFI* Mean fluorescent index. The presence of the metabolic syndrome (MetS) is defined according to the joint interim statement criteria (1).

		**No MetS**	**MetS**	
n		29	35	**p-value**
Total leukocyte count (10^6/ml), (mean (SD))		6.6 (1.6)	6.8 (1.4)	0.69
Total neutrophil count (10^6/ml), (mean (SD))		4.0 (1.4)	3.8 (1.4)	0.69
Total lymphocyte count (10^6/ml), (mean (SD))		2.1 (0.6)	2.2 (0.6)	0.56
Total eosinophil count (10^6ml), (mean (SD))		0.2 (0.1)	0.4 (0.8)	0.2
Total monocyte count (10^6ml), (mean (SD))		0.3 (0.1)	0.4 (0.1)	0.49
Relative CM (%) (mean (SD))		87.0 (4.5)	87.2 (3.2)	0.82
Relative IM (%) (median [IQR])		2.7 [2.2, 3.0]	2.3 [1.9, 2.9]	0.21
Relative NCM (%) (mean (SD))		10.3 (4.3)	10.2 (2.9)	0.89
Absolute number of CM (10^4/ml), (mean (SD))		29.4 (10.5)	31.3 (10.4)	0.48
Absolute number of IM (10^4/ml), (mean (SD))		0.9 (0.4)	0.9 (0.5)	0.97
Absolute number of NSM (10^4/ml), (mean (SD))		3.4 (1.6)	3.6 (1.4)	0.62
MFI CD36	CM (10^3), (mean (SD))	60.5 (22.0)	61.2 (27.4)	0.91
	IM (10^3), (mean (SD))	69.7 (29.4)	68.7 (31.2)	0.9
	NCM (10^3), (mean (SD))	27.8 (17.9)	26.8 (17.3)	0.82
MFI CD64	CM (10^3), (mean (SD))	9.9 (3.9)	9.8 (2.9)	0.96
	IM (10^3), (mean (SD))	7.8 (4.0)	7.6 (2.8)	0.79
	NCM (10^3) (median [IQR])	1.4 [0.9, 2.5]	1.7 [1.0, 2.7]	0.33
MFI CD14	CM (10^3), (mean (SD))	20.7 (3.4)	20.5 (4.2)	0.88
	IM (10^3), (mean (SD))	16.1 (3.4)	16.2 (4.1)	0.99
	NCM (10^3), (mean (SD))	2.7 (1.3)	2.5 (1.3)	0.4
MFI CD16	CM (10^3), (mean (SD))	1.9 (0.6)	1.8 (0.7)	0.77
	IM (10^3), (mean (SD))	15.9 (6.5)	15.4 (5.8)	0.73
	NCM (10^3), (mean (SD))	28.1 (11.2)	27.8 (10.6)	0.92
MFI CD45	CM (10^3), (mean (SD))	6.5 (0.9)	6.5 (1.1)	0.99
	IM (10^3), (mean (SD))	8.6 (1.0)	8.4 (1.1)	0.58
	NCM (10^3), (mean (SD))	7.3 (1.0)	7.5 (0.9)	0.48
MFI HLA-DR	CM (10^3), (mean (SD))	6.9 (3.0)	5.7 (2.4)	0.07
	IM (10^3), (mean (SD))	23.9 (7.5)	20.1 (8.5)	0.07
	NCM (10^3), (mean (SD))	10.3 (3.6)	9.9 (4.4)	0.66
MFI IREM2	*CM (10^3) (median [IQR])*	**2.9 [2.4, 3.4]**	**3.6 [2.8, 5.0]**	**0.02**
	IM (10^3) (median [IQR])	4.5 [4.0, 5.4]	5.8 [4.0, 8.6]	0.08
	NCM (10^3) (median [IQR])	**2.9 [2.6, 3.4]**	**4.1 [2.6, 4.9]**	***0.03***

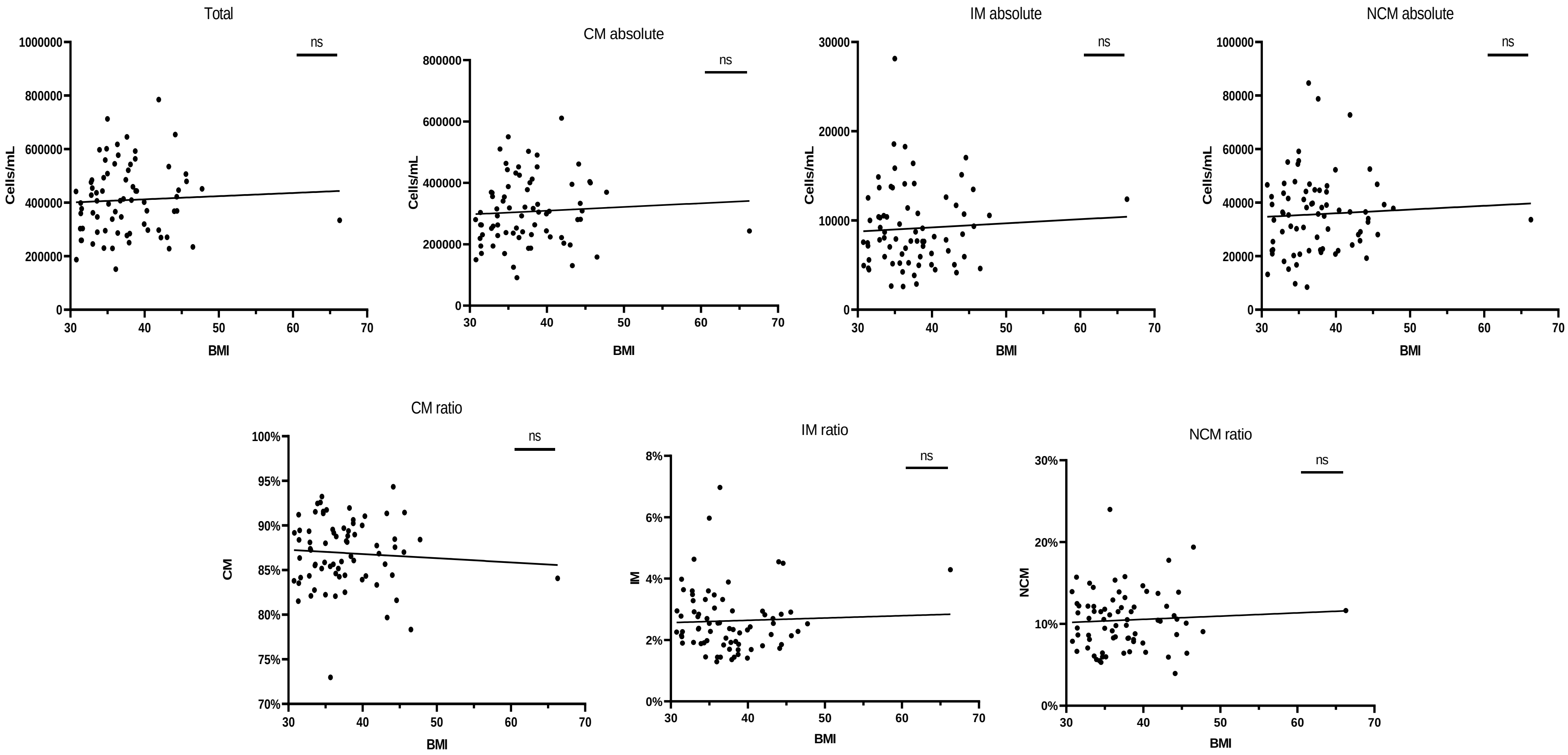

Supplemental figure 4a**:** Body mass index (BMI) does not relate to absolute monocyte counts (upper row) or relative monocytes (lower row) for total monocytes, classical monocytes (CM), intermediate monocytes (IM) or non-classical monocytes (NCM) at baseline. The Pearson correlation was used for all correlations except IM ratio. The Spearman correlation was used for IM ratio. A significance level of $p<0.05$ was used. NS: not significant.

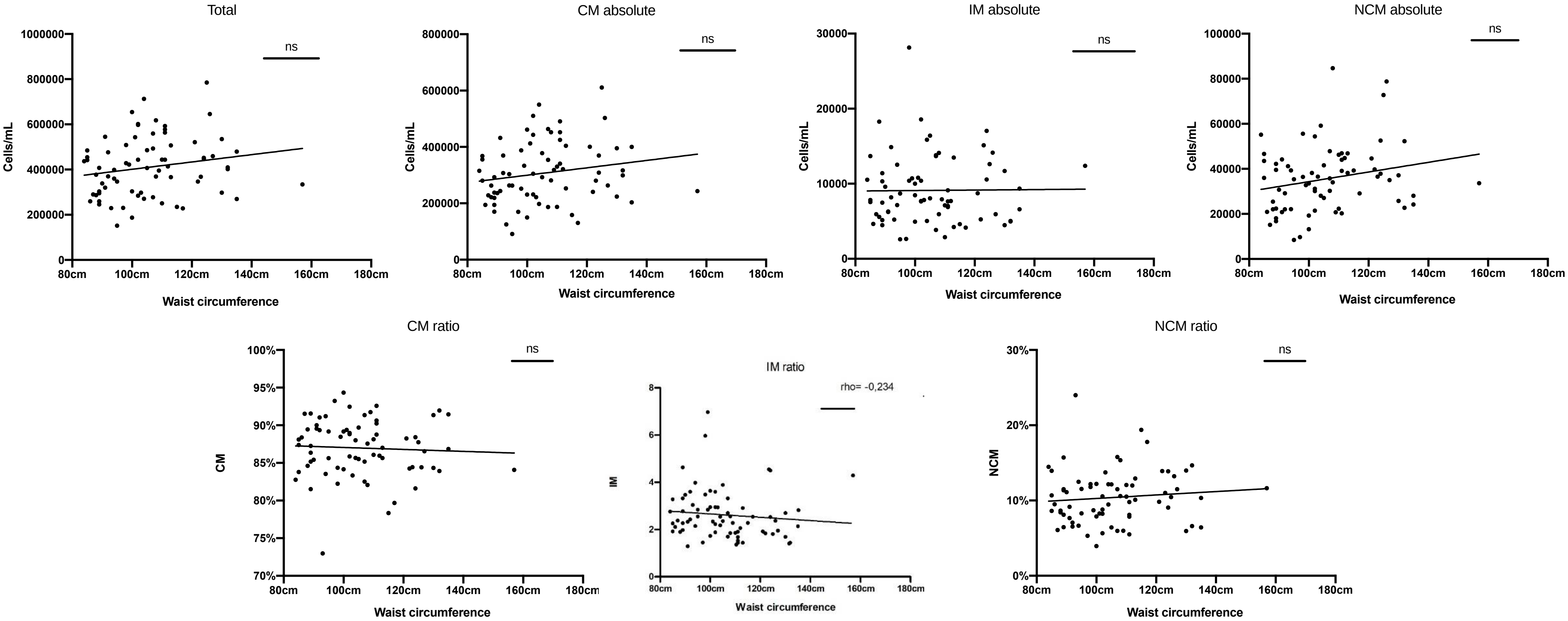

Figure 4b**:** Waist circumference (WC) does not relate to absolute monocyte counts (upper row) or relative monocytes (lower row) for total monocytes, classical monocytes (CM), intermediate monocytes (IM) or non-classical monocytes (NCM) at baseline. The Pearson correlation was used for all correlations except IM ratio. The Spearman correlation was used for IM ratio. All correlations are corrected for age and sex by using a linear regression model. A significance level of $p<0.05$ was used. #not significant after correction, NS: not significant.

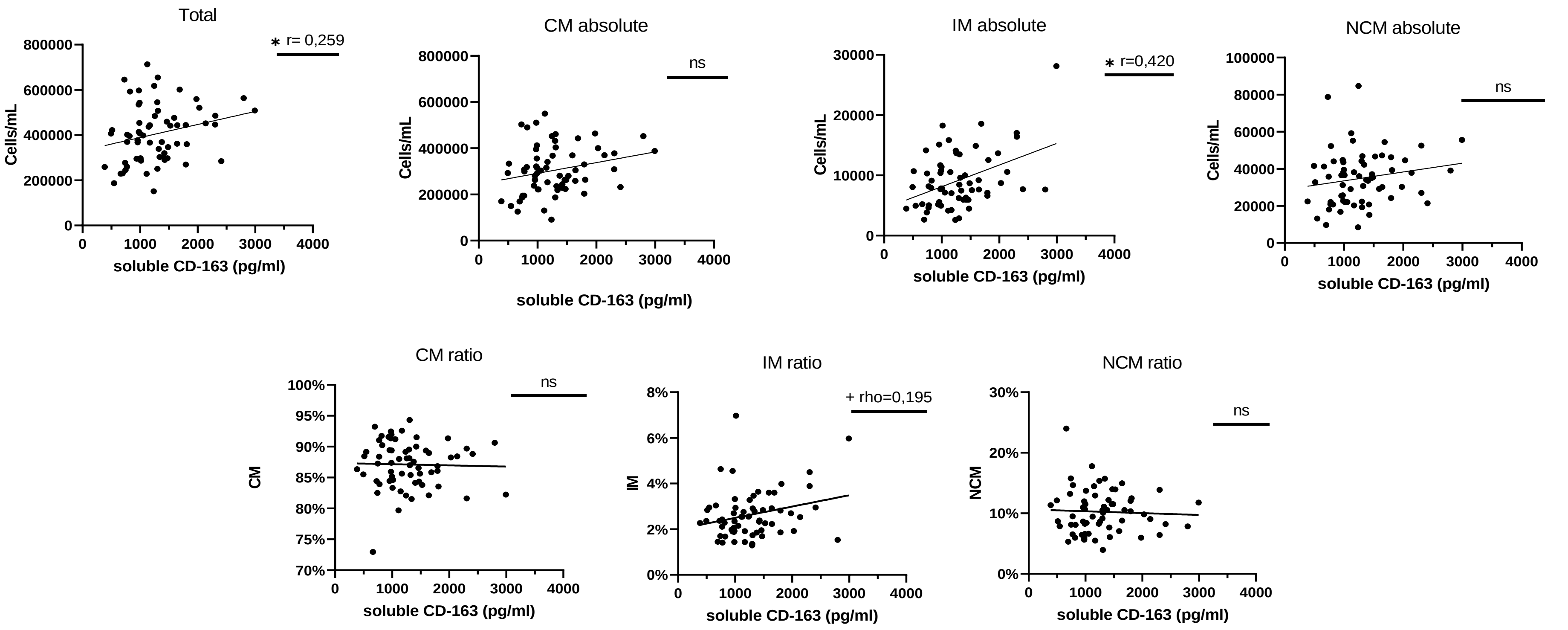

Supplemental figure 4c. sCD163 correlates to total monocyte and intermediate monocyte counts. sCD163 correlations with absolute monocyte counts (upper row) or relative monocytes (lower row) for total monocytes, classical monocytes (CM), intermediate monocytes (IM) or non-classical monocytes (NCM) at baseline. The Pearson correlation was used for all correlations, except IM ratio, and is presented as r=. The Spearman correlation was used for IM ratio and is presented as rho=. All correlations are corrected for age and sex by using a linear regression model. A significance level of $p<0.05$ was used. *: significant before and after correction; +significant after correction; NS not significant

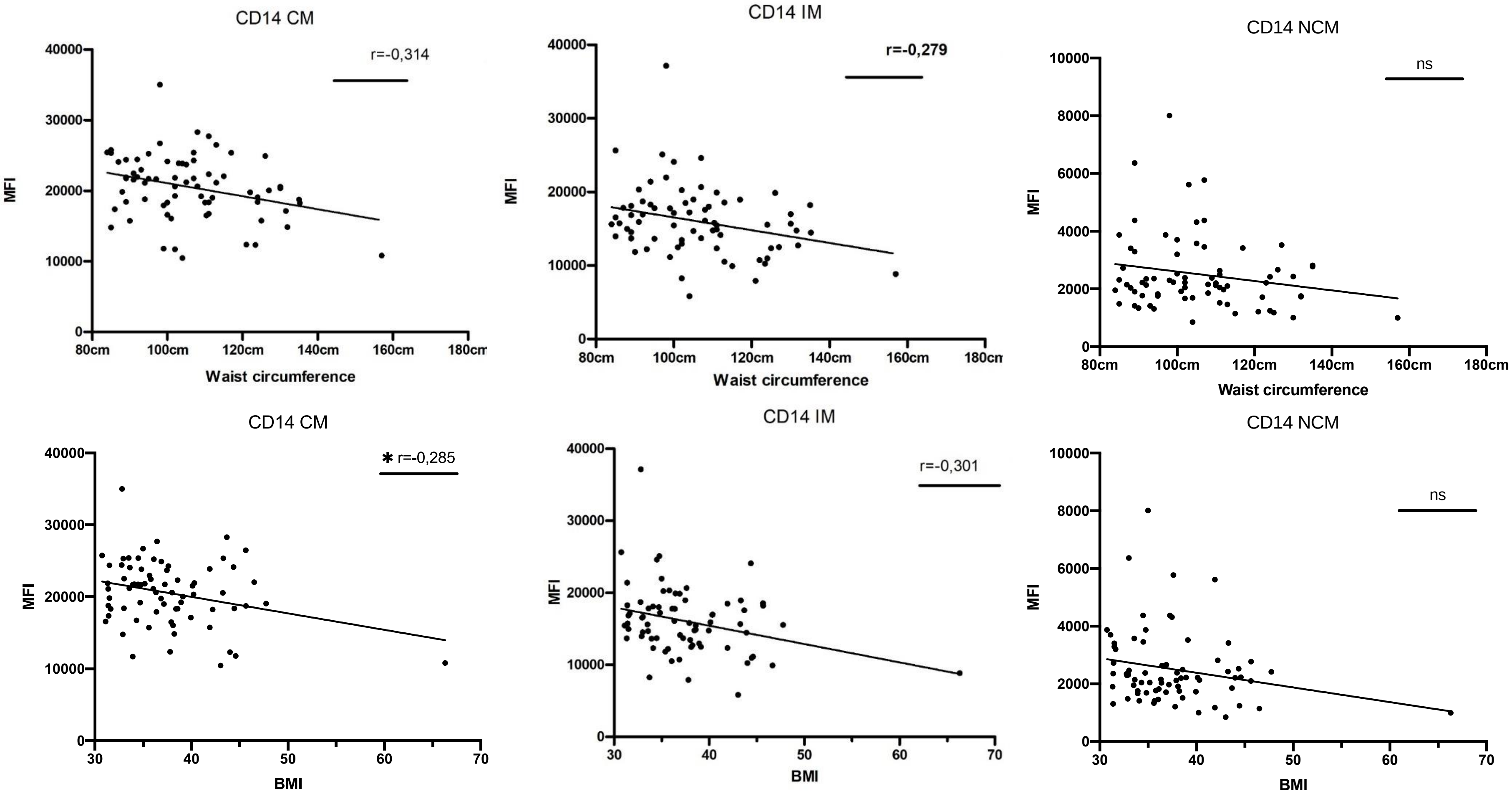

Supplemental figure 4d. **:** CD14 correlations with WC (upper row) or BMI (lower row) in classical monocytes (CM), intermediate monocytes (IM) or non-classical monocytes (NCM) at baseline. The Pearson correlation was used for CD14 expression by CM and IM and is presented as r. The Spearman correlation was used for CD14 expression by NCM. *: significant at $p<0.05$ level; NS not significant.

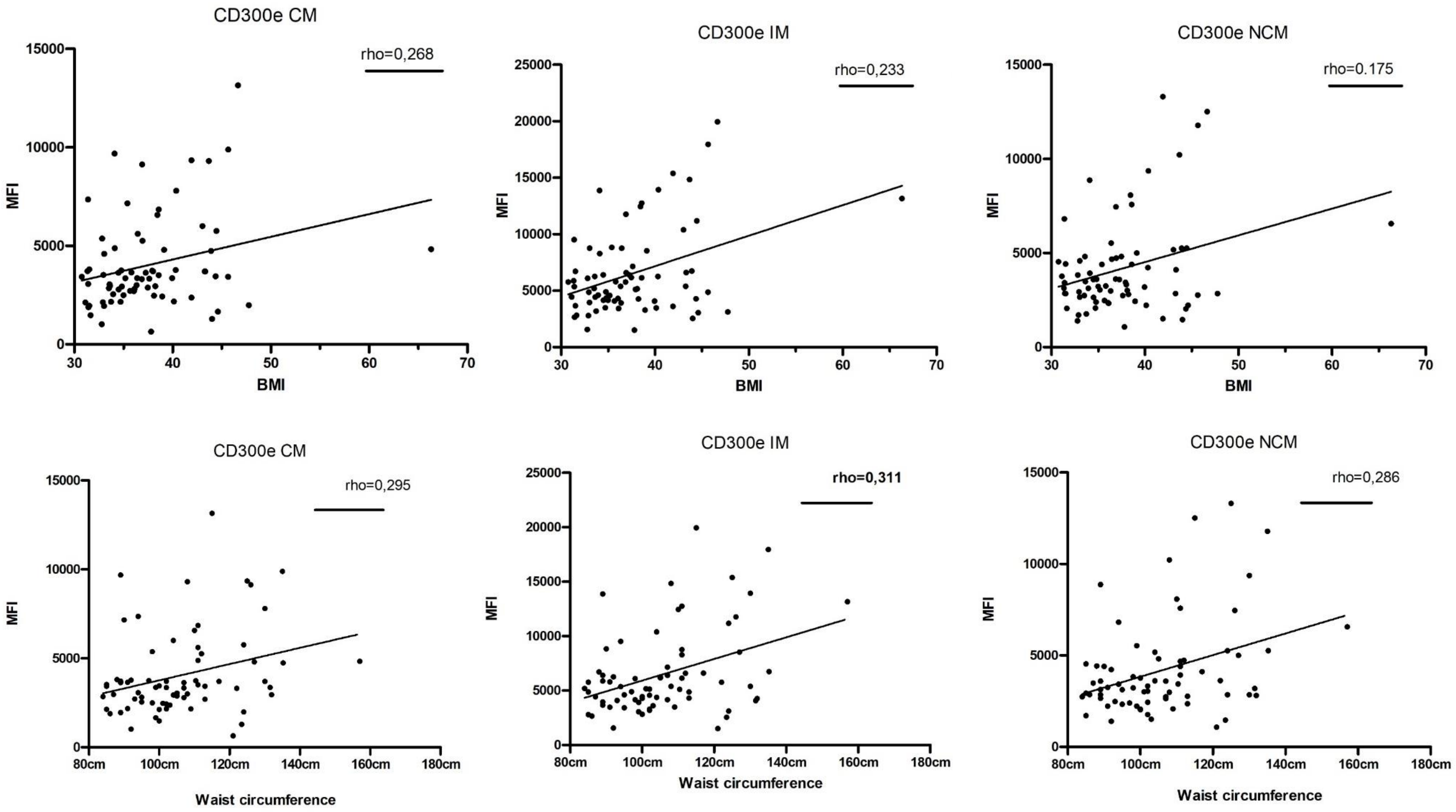

Supplemental figure 4e**:** CD300e correlations with WC (upper row) or BMI (lower row) and classical monocytes (CM), intermediate monocytes (IM) or non-classical monocytes (NCM) at baseline. The Spearman correlation was used for all correlations and is presented as rho. *: significant at p<0.05 level; NS not significant.

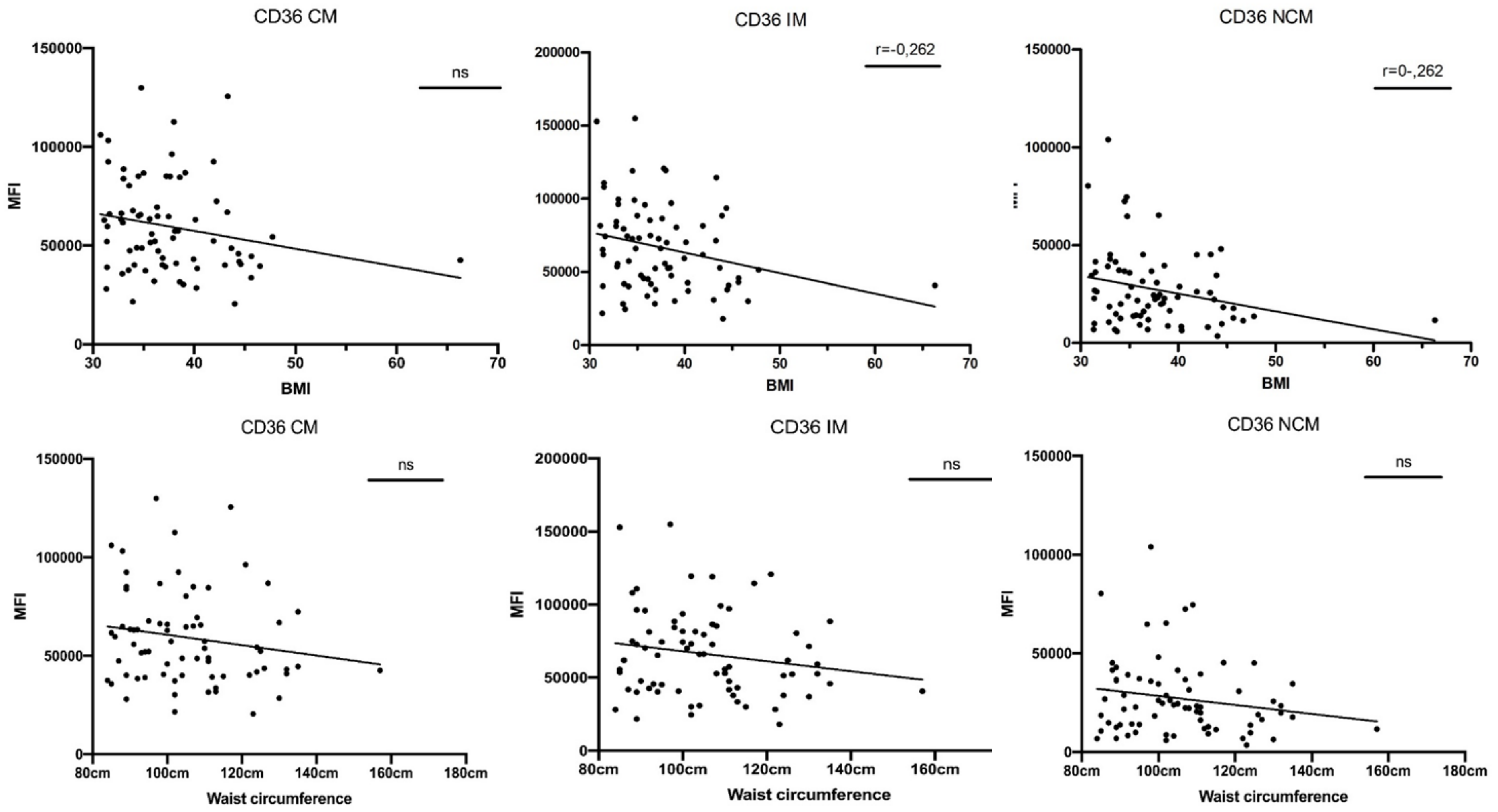

Supplemental figure 4f**:** CD36 correlations with BMI (upper row) or WC (lower row) and classical monocytes (CM), intermediate monocytes (IM) or non-classical monocytes (NCM) at baseline. The Pearson correlation was used for all correlations and is presented as r.
*: significant at p<0.05 level; NS not significant.

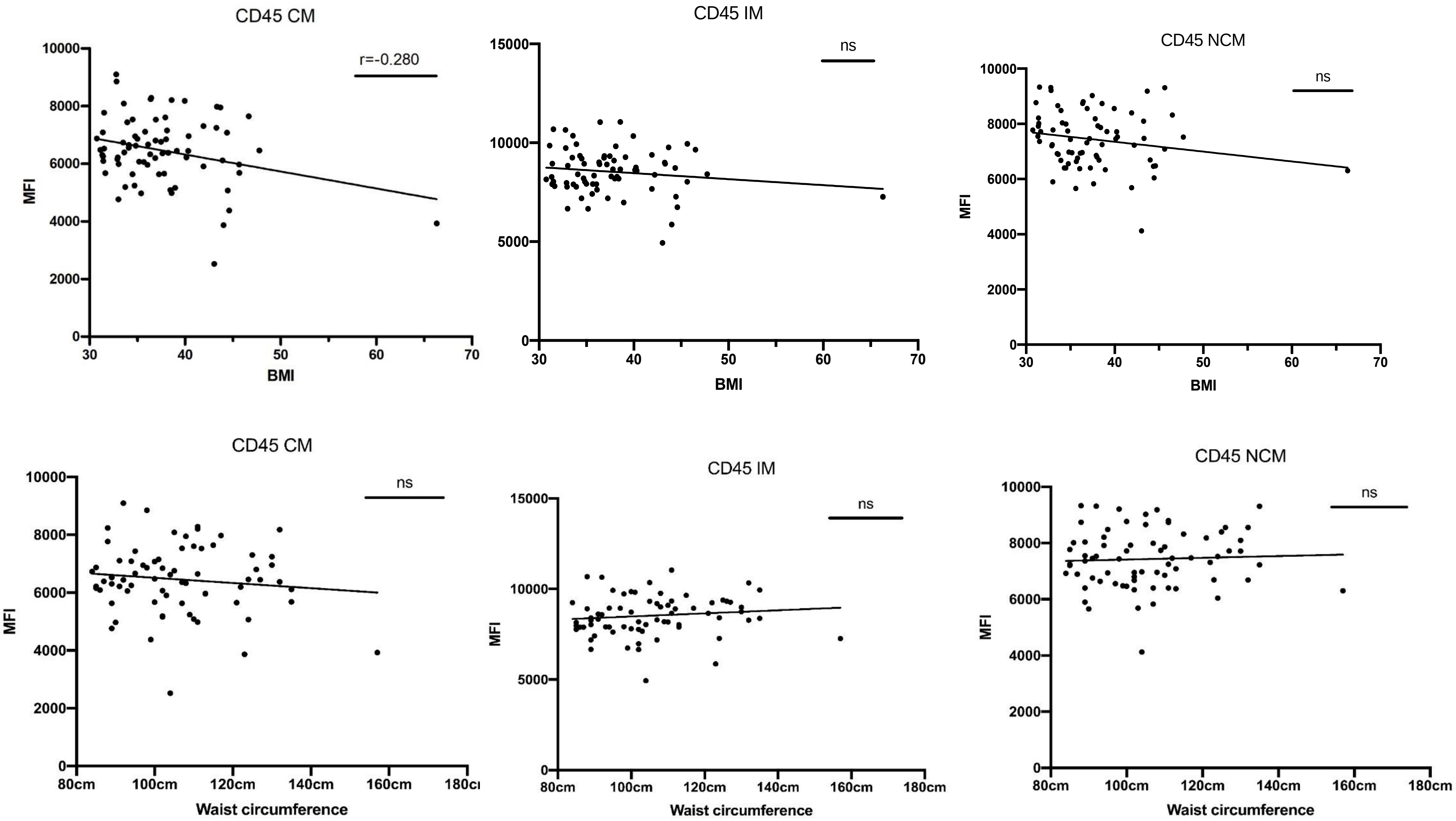

Supplemental figure 4g**:** CD45 correlations with BMI (upper row) or WC (lower row) and classical monocytes (CM), intermediate monocytes (IM) or non-classical monocytes (NCM) at baseline. The Pearson correlation was used for all correlations and is presented as r. *: significant at $p<0.05$ level; NS not significant.

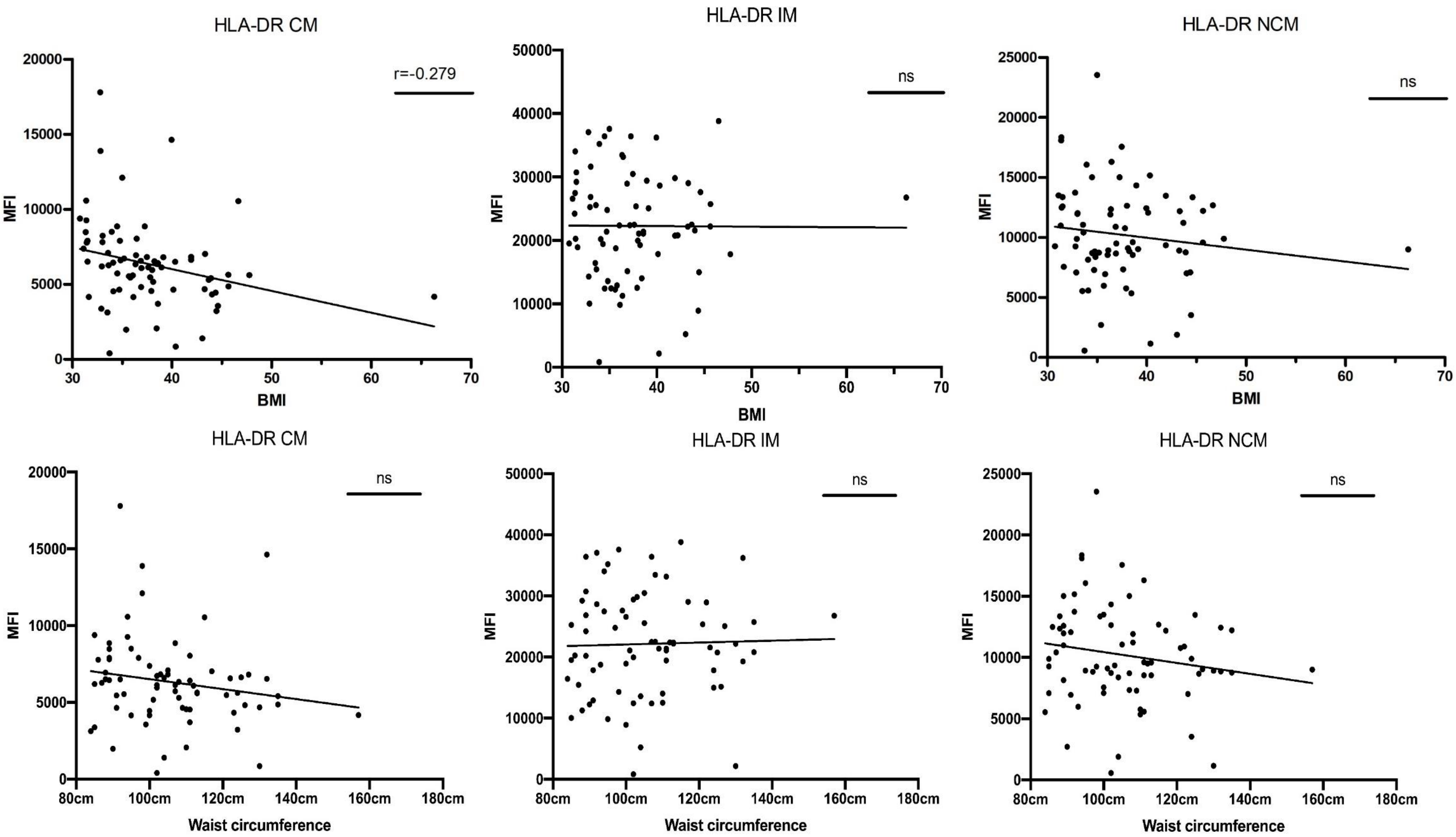

Supplemental figure 4h**:** HLA-DR correlations with BMI (upper row) or WC (lower row) and classical monocytes (CM), intermediate monocytes (IM) or non-classical monocytes (NCM) at baseline. The Pearson correlation was used for all correlations and is presented as r. *: significant at p<0.05 level; NS not significant.

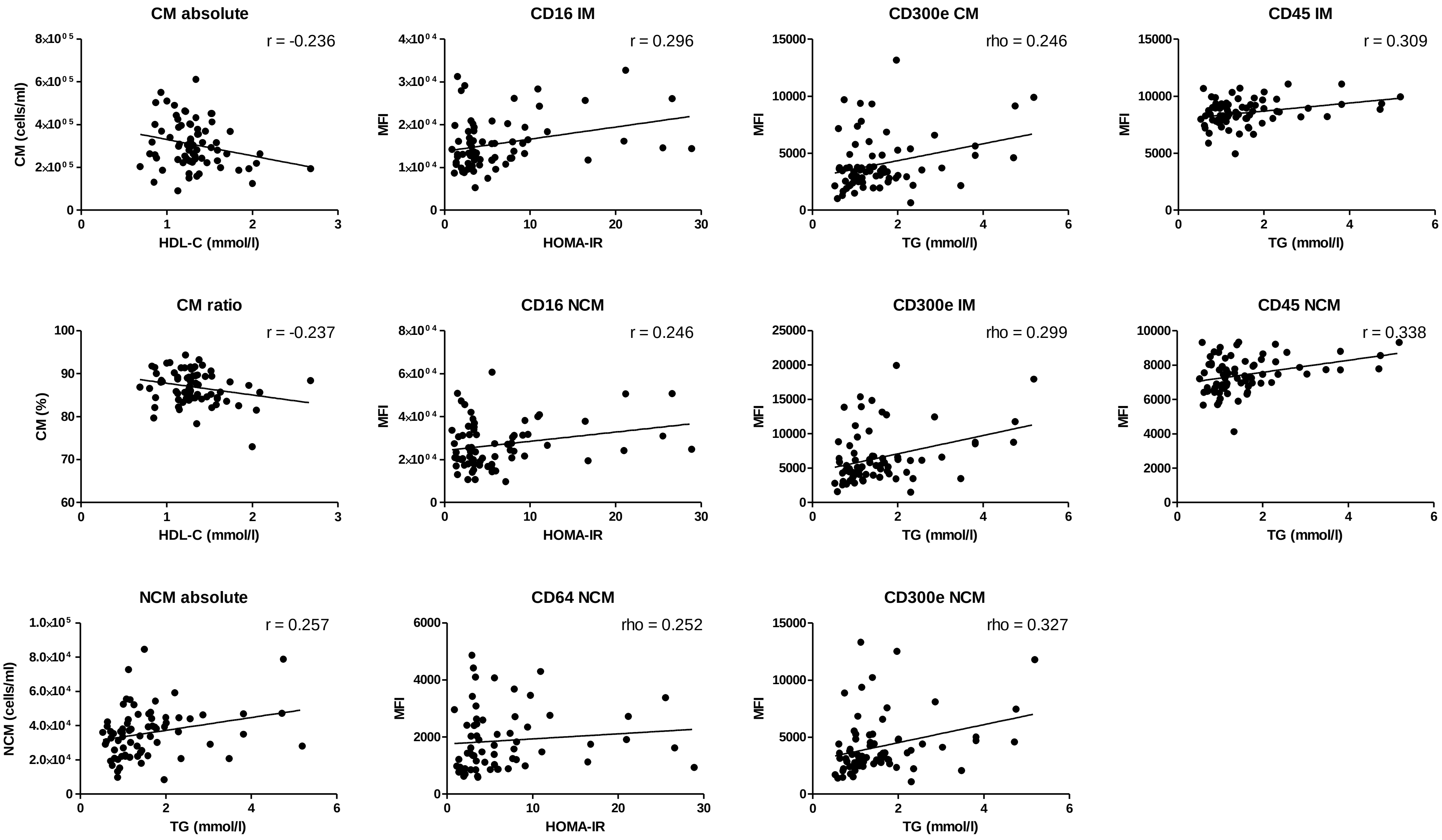

Supplemental data 4i. Significant correlations between metabolic components and monocyte parameters at baseline. All the above correlations are significant at the $p<0.05$ level. Abbreviations: CM *classical monocytes*, IM *intermediate monocytes*, NCM *non-classical monocytes.* HDL-C *high density lipoprotein cholesterol*, TG *triglycerides*, HOMA-IR *Homeostatic model assessment of insulin resistance.* The Pearson correlation was used for all correlations and is presented as r (except for the correlation between CD64 expression and HOMA-IR, and the correlations between CD300e in all subsets with TG, where a spearman correlation was used).

Supplemental data 5.
Improvements in obesity parameters.

	Clinical parameter	n	T0	T1	T2
Participants with complete data at baseline and first evaluation	Self reported daily caloric intake (kcal/day)	30	1734	1696	
	Self reported MET-minutes	20	5086	4706	
Subgroup of participants with complete data at baseline, first and second evaluation	Self reported daily caloric intake (kcal/day)	20	1737	1635	1754
	Self reported increase in MET-minutes	13	4417	4364	5319

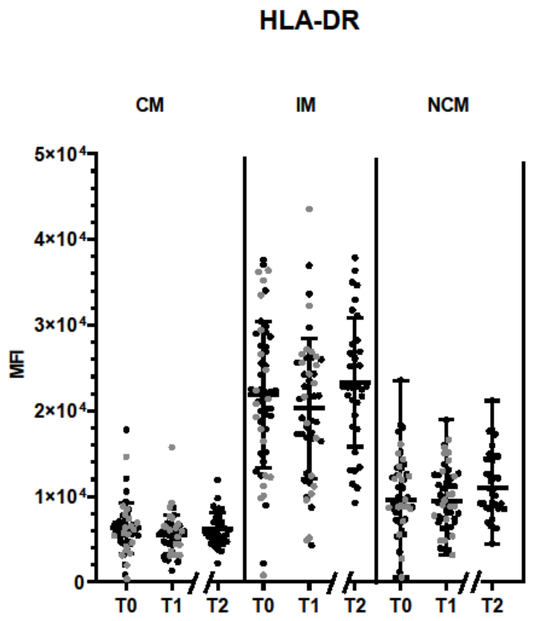

Supplemental data 6. Changes in HLA-DR expression in classical monocytes (CM), intermediate monocytes (IM) and non-classical monocytes (NCM) between T0 and T1 of patients who had a complete follow-up at T1 (N=51) and between T0 and T2 patients who completed the program (n=35). HLA-DR expression by CM and are normally distributed and are presented as mean + SD. HLA-DR expression by NCM is non-normally distributed and presented median + range. Paired T-tests were used to determine the differences between T0 and T1, and between T0 and T2 for normally distributed data, the Wilcoxon signed ranks test for non-normally distributed data. Individuals who had follow-up until T1 are represented as grey dots, individuals with follow-up until T2 are represented as black dots. MFI: median fluorescence intensity. *p<0.05, ** p<0.01 , *** p<0.001.

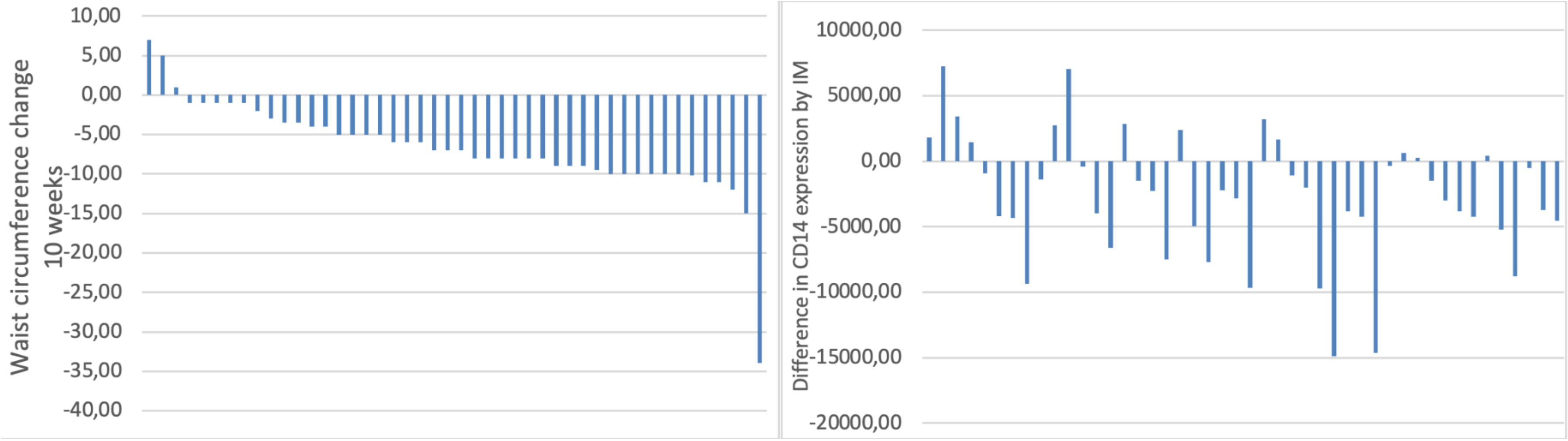

A B

Figure 7: (A) shows all patients, sorted on the absolute decrease in WC in 10 weeks. (B) shows the absolute change in CD14 expression on IM per patient, using the same order as Figure 14A. Pearson's correlation coefficient was 0.325, p=0.023

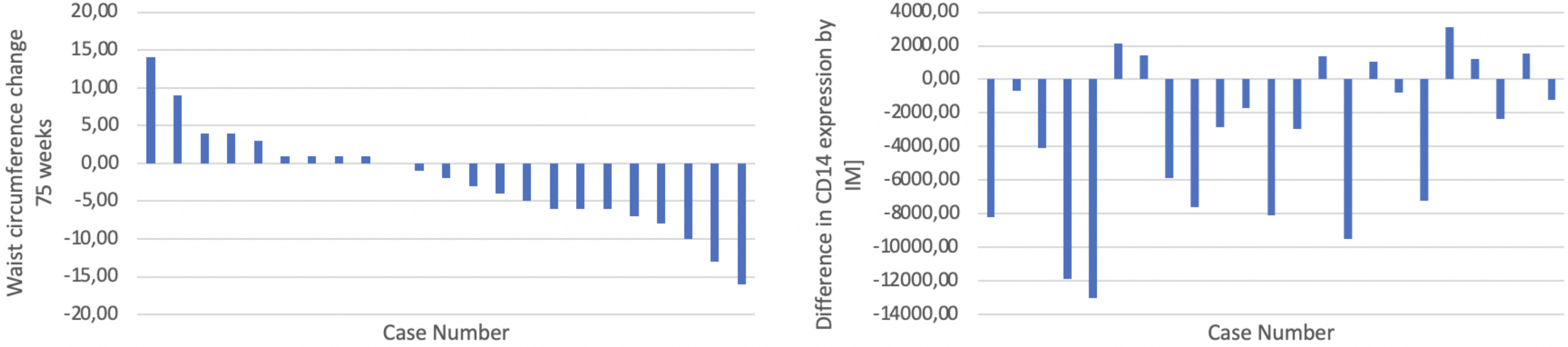

A

B

Figure 8 (A) shows all patients, sorted on the absolute decrease in WC at 1.5 years. (B) shows the absolute change in CD14 expression on IM per patient, using the same order as Figure 15A. Pearson's correlation coefficient was -0.415, p=0.049.

1. Alberti KG, Eckel RH, Grundy SM, Zimmet PZ, Cleeman JI, Donato KA, et al. Harmonizing the metabolic syndrome: a joint interim statement of the International Diabetes Federation Task Force on Epidemiology and Prevention; National Heart, Lung, and Blood Institute; American Heart Association; World Heart Federation; International Atherosclerosis Society; and International Association for the Study of Obesity. Circulation. 2009;120(16):1640-5.
